# A Variational Approach to Video Registration with Subspace Constraints

**DOI:** 10.1007/s11263-012-0607-7

**Published:** 2013-04-02

**Authors:** Ravi Garg, Anastasios Roussos, Lourdes Agapito

**Affiliations:** Queen Mary University of London, Mile End Road, London, E1 4NS UK

## Abstract

This paper addresses the problem of non-rigid video registration, or the computation of optical flow from a reference frame to each of the subsequent images in a sequence, when the camera views deformable objects. We exploit the high correlation between 2D trajectories of different points on the same non-rigid surface by assuming that the displacement of any point throughout the sequence can be expressed in a compact way as a linear combination of a low-rank motion basis. This subspace constraint effectively acts as a trajectory regularization term leading to temporally consistent optical flow. We formulate it as a robust soft constraint within a variational framework by penalizing flow fields that lie outside the low-rank manifold. The resulting energy functional can be decoupled into the optimization of the brightness constancy and spatial regularization terms, leading to an efficient optimization scheme. Additionally, we propose a novel optimization scheme for the case of vector valued images, based on the dualization of the data term. This allows us to extend our approach to deal with colour images which results in significant improvements on the registration results. Finally, we provide a new benchmark dataset, based on motion capture data of a flag waving in the wind, with dense ground truth optical flow for evaluation of multi-frame optical flow algorithms for non-rigid surfaces. Our experiments show that our proposed approach outperforms state of the art optical flow and dense non-rigid registration algorithms.

## Introduction

**Fig. 1:**
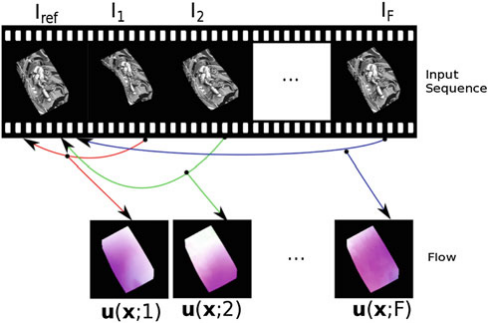
Video registration is equivalent to the problem of estimating dense optical flow $\varvec{u}(\varvec{x};n)$ between a reference frame $I_{ref}$ and each of the subsequent frames $I_n$ in a sequence. We propose a multi-frame optical flow algorithm that exploits temporal consistency by imposing subspace constraints on the 2D image trajectories

Optical flow in the presence of non-rigid deformations is a challenging task and an important problem that continues to attract significant attention from the computer vision community. It has wide ranging applications from medical imaging and video augmentation to non-rigid structure from motion. Given a template image of a non-rigid object and an input image of it after deforming, the task can be described as one of finding the displacement field (warp) that relates the input image back to the template. In this paper we consider long video sequences instead of a single pair of frames—each of the images in the sequence must be aligned back to the reference frame. Our work concerns the estimation of the vector field of displacements that maps pixels in the reference frame to each image in the sequence (see Fig. 1).

**Fig. 2:**
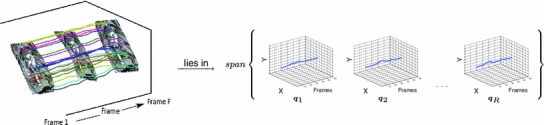
The strong correlation between 2D trajectories of different points on the same non-rigid surface can be exploited to impose temporal coherence by modelling long term temporal coherence imposing subspace constraints. These trajectories lie on a lower dimensional manifold which leads to a significant reduction in the dimensionality of the problem while implicitly imposing some form of temporal smoothness

Two significant difficulties arise. First, the image displacements between the reference frame and subsequent ones are large since we deal with long sequences. Secondly, as a consequence of the non-rigidity of the motion, multiple warps can explain the same pair of images causing ambiguity. In this paper we show that a multi-frame approach allows us to exploit temporal information, resolving these ambiguities and improving the overall quality of the optical flow. We make use of the strong correlation between 2D trajectories of different points on the same non-rigid surface. These trajectories lie on a lower dimensional subspace and we assume that the trajectory vector storing 2D positions of a point across time can be expressed compactly as a linear combination of a low-rank motion basis. This leads to a significant reduction in the dimensionality of the problem while implicitly imposing some form of temporal smoothness. Figure 2 depicts the lower dimensional trajectory subspace.

Subspace constraints have been used before both in the context of sparse point tracking (Irani 2002; Brand 2001; Torresani et al. 2001; Torresani and Bregler 2002) and optical flow (Irani 2002; Garg et al. 2010) in the rigid and non-rigid domains, to allow correspondences to be obtained in low textured areas. While Irani’s original rigid (Irani 2002) formulation along with its non-rigid extensions (Torresani et al. 2001; Brand 2001; Torresani and Bregler 2002) relied on minimizing the linearized brightness constraint without smoothness priors, Garg et al. (2010) extended the subspace constraints to the continuous domain in the non-rigid case using a variational approach. Nir et al. (2008) propose a variational approach to optical flow estimation based on a spatio-temporal model. However, all of the above approaches impose the subspace constraint as a hard constraint. Hard constraints are vulnerable to noise in the data and can be avoided by substituting them with principled robust constraints.In this paper we extend the use of multi-frame temporal smoothness constraints within a variational framework by providing a more principled energy formulation with a robust soft constraint which leads to improved results. In practice, we penalize deviations of the optical flow trajectories from the low-rank subspace manifold, which acts as a temporal regularization term over long sequences. We then take advantage of recent developments (Chambolle 2004; Chambolle and Pock 2011) in variational methods and optimize the energy defining a variant of the duality-based efficient numerical optimization scheme. We are also able to prove that our soft constraint is preferable to a hard constraint imposed via reparameterization. To do this we provide a formulation of the hard constraint and its optimization and we perform thorough experimental comparisons where we show that the results obtained via the soft constraint always outperform those obtained after reparameterization.

The paper is organized as follows. In Sect. 2 we describe related approaches and discuss the contributions of our work. Section 3 defines the trajectory subspace constraints that we use in our formulation. In Sect. 4 we describe the energy and provide a discussion on the design of our effective trajectory regularizer. Section 5 addresses the optimization of our proposed energy. This is followed by a description of the estimation of the motion basis in Sect. 6. In Sect. 7 we propose the extension of our algorithm to vector-valued images and Sect. 8 discusses implementation details. Finally Sect. 9 describes the alternative formulation of the subspace constraint as a hard constraint while Sect. 10 describes our experimental evaluation.

## Related Work and Contribution

Variational methods formulate the optical flow or image alignment problems as the optimization of an energy functional in a continuous domain. Stemming from Horn and Schunck’s original approach (Horn and Schunck 1981), the energy incorporates a data term that accounts for the brightness constancy assumption and a regularization term that allows to fill-in flow information in low textured areas. Variational methods have seen a huge surge in recent years due to the development of more sophisticated and robust data fidelity terms which are robust to changes in image brightness or occlusions (Brox and Malik 2011; Brox et al. 2004); the addition of efficient regularization terms such as Total Variation (TV) (Zach et al. 2007; Wedel et al. 2008) or temporal smoothing terms (Weickert and Schnörr 2001b); and new optimization strategies that allow computation of highly accurate (Wedel et al. 2009) and real time optical flow (Zach et al. 2007) even in the presence of large displacements (Alvarez et al. 2000; Brox and Malik 2011; Steinbruecker et al. 2009).

One important recent advance in variational optical flow methods has been the development of the duality based efficient optimization of the so-called TV-$\mathbf{L}^1$ formulation (Zach et al. 2007; Chambolle and Pock 2011) (which owes its name to the Total Variation that is used for regularization and the robust $\mathbf{L}^1$-norm that is used in the data fidelity term). An example of this class is the Improved TV-$\mathbf{L}^1$ (ITV-$\mathbf{L}^1$) method (Wedel et al. 2009), which yielded notable quantitative performance, by also carefully considering some practical aspects of the optical flow algorithm.Duplication of the optimization variable via a quadratic relaxation is used to decouple the linearized data and regularization terms, decomposing the optimization problem into two, each of which is a convex energy that can be solved in a globally optimal manner. The minimization algorithm then alternates between solving for each of the two variables assuming the other one fixed. One of the key advantages of this decoupling scheme is that since the data term is *point-wise* independent, its optimization can be highly parallelized using graphics hardware (Zach et al. 2007). Following its success in optical flow computation, this optimization scheme has since been successfully applied to motion and disparity estimation (Pock et al. 2010) and real time dense 3D reconstruction (Newcombe et al. 2011; Stuehmer et al. 2010). In this work we adopt this efficient duality based TV-$\mathbf{L}^1$ optimization scheme (Zach et al. 2007) and extend it to the case of multi-frame optical flow for video registration, by modelling long term temporal coherence imposing subspace constraints.

Despite being such a powerful cue most optical flow algorithms do not take advantage of temporal coherence and only work on pairs of images. Few previous attempts to multi-frame optical flow estimation exist in the literature (Weickert and Schnörr 2001b, a; Papadakis et al. 2007; Nir et al. 2008; Werlberger et al. 2009; Volz et al. 2011). Even in those cases, temporal smoothness constraints are only exploited over a very small number of frames (typically $1$ or $2$ frames either side of the current image) and not for an entire sequence. This is mostly due to the difficulty of providing an explicit model for longer term trajectories. In recent work Volz et al. (2011) report improvements in optical flow computation by imposing first and second order trajectory smoothness over $5$ frames. We take this further and exploit temporal coherence throught the entire video. Moreover, while previous approaches incorporate explicit temporal smoothness regularization terms over a few frames, our subspace constraint acts as an implicit long term trajectory regularization term leading to temporally consistent optical flow.

Our approach is related to the recent work of Garg et al. (2010) in which dense multi-frame optical flow for non-rigid motion is computed under hard subspace constraints. Our approach departs in a number of ways. First, while Garg et al. (2010) imposes the subspace constraint via reparameterization of the optical flow, we use a soft constraint and optimize over two sets of closely coupled flows, one that lies on the low-rank manifold and one that does not. Secondly, our use of a robust penalizer for the data term allows us to have more resilience than Garg et al. (2010) against occlusions and appearance changes. Moreover, our use of a modified Total Variation regularizer instead of the non-robust $\mathbf{L}^2$-norm and quadratic regularizer used by Garg et al. (2010) allows to preserve object boundaries. Finally, by providing a generalization of the subspace constraint, we have extended the approach to deal with any orthonormal basis and not just the PCA basis. More recently Ricco and Tomasi (2012) also proposed the use of subspace constraints to model multi-frame optical flow with explicit reasoning for occlusions. However, their approach is restricted to hard subspace constraints with a known PCA basis which is computed from sparse feature tracking.

Non-rigid image registration, has recently seen substantial progress in its robust estimation in the case of severe deformations and large baselines both from keypoint-based and learning based approaches. Successful keypoint-based approaches to deformable image registration include the parametric^1^ approach of Pizarro and Bartoli (2010) who propose a warp estimation algorithm that can cope with wide baseline and self-occlusions using a piecewise smoothness prior on the deforming surface. A direct approach that uses all the pixels in the image is used as a refinement step. Discriminative approaches on the other hand, learn the mapping that predicts the deformation parameters given a distorted image but require a large number of training samples. In recent work, Tian and Narasimhan (2010) combine generative and discriminative approaches which results in lowering the total number of training samples.

### Our contribution

In this paper we adopt a robust approach to non-rigid image alignment where instead of imposing the hard constraint that the optical flow must lie on the low-rank manifold (Garg et al. 2010), we penalize flow fields that lie outside it. Formulating the manifold constraint as a *soft constraint* using variational principles (Garg et al. 2011) leads to an energy with a quadratic term that allows us to adopt a decoupling scheme, related to the one described above (Zach et al. 2007; Chambolle and Pock 2011), for its efficient optimization. We propose a new *anisotropic trajectory regularization* term, parameterized in terms of the basis coefficients, instead of the full flow field. This results in an important dimensionality reduction in this term, which is usually the bottleneck of other quadratic relaxation duality based approaches (Zach et al. 2007; Chambolle and Pock 2011). Moreover, the optimization of our regularization step can be highly parallelized due to the independence of the orthonormal basis coefficients adding further advantages to previous approaches. Our approach can be seen as an extension of Zach et al. (2007) efficient TV-$\mathbf{L}^1$ flow estimation algorithm to the case of multi-frame non-rigid optical flow, where the addition of subspace constraints acts as a temporal regularization term. In practice, our approach is equivalent to Zach et al. (2007) in the degenerate case where the identity matrix is chosen as the motion basis.

We take advantage of the high level of parallelism inherent to our approach by developing a GPU implementation using the Nvidia CUDA framework. This parallel implementation vastly outperforms the equivalent Matlab code.

Additionally, we provide an extension of our multi-frame approach to the case of vector-valued images which allows us to use the information from all colour channels in image sequences, and further improve results. Our novel optimization scheme is based on the dualization of the linearized data term. Unlike Râket et al.’s previous attempt to extend TV-$\mathbf{L}^1$ flow to vector valued images (Rakêt et al. 2011), our new algorithm is not restricted to the use of the $\mathbf{L}^1$-norm penaliser and instead allows the use of more general convex robust penalizers in the data term.

Currently, there are no benchmark datasets for the evaluation of optical flow that include long sequences of non-rigid deformations. In particular, the most popular one (Baker et al. 2011) (Middlebury) does not incorporate any such sequences. To facilitate the quantitative evaluation of multi-frame non-rigid registration and optical flow and to promote progress in this area, we provide a new dataset based on motion capture data of a flag waving in the wind, with dense ground truth optical flow.

Our quantitative evaluation on this dataset using different motion bases shows that our proposed approach improves on state of the art algorithms including large displacement (Brox and Malik 2011) and duality based (Zach et al. 2007) optical flow algorithms and the parametric dense non-rigid registration approach of Pizarro and Bartoli (2010).

## Multi-frame Image Registration

Consider a video sequence of non-rigid objects moving and deforming in 3D. In the classical optical flow problem, one seeks to estimate the vector field of image point displacements independently for each pair of consecutive frames. In this paper, we adopt the following multi-frame reformulation of the problem. Taking one frame as the reference template, typically the first frame, our goal is to estimate the 2D trajectories of every point visible in the reference frame over the entire sequence, using a multi-frame approach (Fig. 1 illustrates our approach). The use of temporal information in this way allows us to predict the location of points not visible in a particular frame making us robust to self-occlusions or external occlusions by other objects.

### Low-Rank Trajectory Space

To solve the multi-frame optical flow problem, we make use of the fact that the 2D image trajectories of points on an object are highly correlated, even when the object is deforming. We model this property by assuming that the trajectories lie near a low-dimensional linear subspace. This assumption is analogous to the non-rigid low-rank shape model, first proposed by Bregler et al. (2000), which states that the time varying 3D shape of a non-rigid object can be expressed as a linear combination of a low-rank shape basis. This rank constraint has been successfully exploited for 3D reconstruction by Non-Rigid Structure from Motion (NRSfM) algorithms (Torresani et al. 2008) where the matrix of 2D tracks is factorized into the product of two low-rank matrices: a motion matrix that describes the camera pose and time varying coefficients and a shape matrix that encodes the basis shapes.

The low-rank shape basis model of Bregler et al. (2000), Torresani et al. (2008) exploits the spatial properties of non-rigid motion, introducing rank constraints on the 3D location of the set of points (shape) at any given frame. Interestingly, the dual formulation of this model states that the rank constraint can be instead applied to the trajectories of each individual point, modelling them as a linear combination of *basis trajectories*. Therefore, the motion and shape matrices can exchange their roles as basis and coefficients and we can either interpret the 2D tracks as the projection of a linear combination of 3D basis shapes or as the linear combination of a 2D motion basis. This concept of non-rigid *trajectory basis* was first introduced in 2D by Torresani and Bregler (2002) who applied it to non-rigid 2D tracking as an extension of the rigid subspace constraints proposed by Irani (2002). Later Akhter et al. (2008, 2011) extended the *trajectory basis* to 3D to model non-rigid 3D trajectories using the Discrete Cosine Transform (DCT) basis.

### Dense Trajectory Subspace Constraints

This paper extends the use of 2D trajectory subspace constraints to the case of estimating dense multi-frame optic flow using a variational approach.

More precisely, we assume that the input image sequence has $F$ frames and the $n_0$-th frame, $n_0 \in \{1,\ldots ,F\}$ has been chosen as the reference. We denote by $\Omega \subset \mathbb{R }^2$ the image domain and we define the function:

1 $$\begin{aligned} \varvec{u}(\varvec{x};n)= \left[ \begin{array}{c} u_1(\varvec{x};n)\\ u_2(\varvec{x};n)\\ \end{array} \right] :\Omega \times \{1,\ldots ,F\} \rightarrow \mathbb{R }^2 \end{aligned}$$

that represents the point trajectories in the following way. For every visible point $\varvec{x}\in \Omega$ in the reference image, $\varvec{u}(\varvec{x};\cdot ): \{1,\ldots ,F\} \rightarrow \mathbb{R }^2$ is its discrete-time 2D trajectory over all frames of the sequence. The coordinates of each trajectory $\varvec{u}(\varvec{x};\cdot )$ are expressed with respect to the position of the point $\varvec{x}$ at $n=n_0,$ which means that $\varvec{u}(\varvec{x};n_0)=0$ and that the location of the same point in frame $n$ is $\varvec{x}+\varvec{u}(\varvec{x};n).$We use the term *multi-frame optical flow* to describe $\varvec{u},$ since it corresponds to a multi-frame extension of the conventional optical flow: the latter is given by $\varvec{u}(\varvec{x};2)$ in the degenerate case where the sequence contains only $F=2$ frames and the first one is considered as the reference ($n_0=1$).

Mathematically, the robust linear subspace constraint on the 2D trajectories $\varvec{u}(\varvec{x};n)$ can be expressed in the following way. For all $\varvec{x}\in \Omega$ and $n \in \{1,\ldots ,F\}$:

2 $$\begin{aligned} \varvec{u}(\varvec{x}; n) = \sum _{i=1}^R \varvec{q}_i(n) L_i(\varvec{x}) \,\,+\,\, \varvec{\varepsilon }(\varvec{x}; n), \end{aligned}$$

which states that the trajectory $\varvec{u}(\varvec{x};\cdot )$ of any point $\varvec{x}\in \Omega$ can be approximated as the linear combination of $R$ basis trajectories $\varvec{q}_1(n),\ldots ,\varvec{q}_R(n): \{1,\ldots ,F\} \rightarrow \mathbb{R }^2$ that are independent from the point location. We include a modeling error $\varvec{\varepsilon }(\varvec{x}; n)$ which will allow us to impose the subspace constraint as a penalty term.Normally the values of $\varvec{\varepsilon }(\varvec{x}; n)$ are relatively small, yet sufficient to improve the robustness of the multi-frame optical flow estimation.

**Fig. 3:**
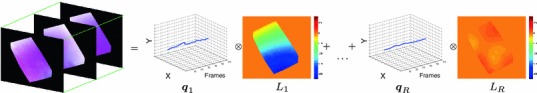
The displacement of any point throughout the sequence can be expressed in a compact way as a linear combination of a low-rank trajectory basis. The basis vectors $\varvec{q}_i(n)$ encode the temporal information while the coefficient maps $\varvec{L}_i$ describe the spatial distribution of the individual basis trajectories

Note that we consider that the chosen trajectory basis is *orthonormal*. We refer to the linear span of these basis trajectories as a *trajectory subspace* and denote it by $\mathcal{S }_Q.$ The linear combination is controlled by coefficients $L_i(\varvec{x})$ that depend on $\varvec{x},$ therefore we can interpret the collection of all the coefficients for all the points $\varvec{x}\in \Omega$ as a vector-valued image $\varvec{L}(\varvec{x})\triangleq [L_1(\varvec{x}),\ldots ,L_R(\varvec{x})]^T : \Omega \rightarrow \mathbb{R }^R.$ Figure 3 illustrates the subspace constraint.

In many cases, effective choices for the model order (or rank) $R$ correspond to values smaller than $2F,$ which means that the above representation is compact and achieves a significant dimensionality reduction on the point trajectories.

We now re-write equation (2) in matrix notation, which will be useful in the subsequent presentation. Let $\varvec{\mathcal{U }}(\varvec{x})$ and $\varvec{\mathcal{E }}(\varvec{x})$ $: \Omega \rightarrow \mathbb{R }^{2F}$ be equivalent representations of the functions $\varvec{u}(\varvec{x};n)$ and $\varvec{\varepsilon }(\varvec{x};n)$ that are derived by vectorizing the dependence on the discrete time $n$ and let $\mathrm{Q}$ be the trajectory basis matrix whose columns contain the basis elements $\varvec{q}_1(n),\ldots ,\varvec{q}_R(n),$ after vectorizing them in the same way:

3 $$\begin{aligned}&\underbrace{\varvec{\mathcal{U }}}_{2F\times 1}(\varvec{x}) \triangleq \left[ \! \begin{array}{c} \varvec{u}(\varvec{x};1) \\ \vdots \\ \varvec{u}(\varvec{x};F) \\ \end{array} \!\right] , \,\, \underbrace{\mathrm{Q}}_{2F\times R} \triangleq \left[ \! \begin{array}{ccc} \varvec{q}_1(1) &{} \cdots &{} \varvec{q}_R(1) \\ \vdots &{} &{} \vdots \\ \varvec{q}_1(F) &{} \cdots &{} \varvec{q}_R(F) \\ \end{array} \right] , \,\, \nonumber \\&\underbrace{\varvec{\mathcal{E }}}_{2F\times 1}(\varvec{x}) \triangleq \left[ \begin{array}{c} \varvec{\varepsilon }(\varvec{x};1) \\ \vdots \\ \varvec{\varepsilon }(\varvec{x};F) \\ \end{array} \right] \end{aligned}$$

The subspace constraint (2) can now be written as follows:

4 $$\begin{aligned} \varvec{\mathcal{U }}(\varvec{x}) = \mathrm{Q} \, \varvec{L}(\varvec{x}) \,\,+\,\, \varvec{\mathcal{E }}(\varvec{x}), \, \forall \varvec{x}\in \Omega \end{aligned}$$

### Non-Rigid Video Registration from Multi-frame Optical Flow

Let $I(\varvec{x};n):\Omega \times \{1,\ldots ,F\} \rightarrow \mathbb{R }$ be the sequence of grayscale image frames, which are given either directly from the input frames or from the input frames after some preprocessing, such as structure-texture decomposition (Wedel et al. 2009).

In our formulation, the estimation of the multi-frame optical flow is equivalent to the simultaneous registration of all the frames with the reference frame $n_0$: Recall that for every frame $n$ the coordinates $\varvec{x}+\varvec{u}(\varvec{x};n)$ yield the current location of any image point $\varvec{x}$ of the reference. Therefore, the image:

5 $$\begin{aligned} \varvec{x}\rightarrow I\left( \varvec{x}+\varvec{u}(\varvec{x};n) \,\,;\,\, n \right) \end{aligned}$$

is the registered version of the image $I(\varvec{x}\,\,;\,\, n)$ back to the reference $I(\varvec{x}\,\,;\,\, n_0),$ or in other words it is the *warping* of the image $I(\varvec{x}\,\,;\,\, n)$ to the image $I(\varvec{x}\,\,;\,\, n_0).$ As it will be described later, we expect that the brightness differences between every registered image and the reference image to be small and therefore we use an appropriate brightness constancy term in our proposed energy.

## Variational Multi-frame Optical Flow Estimation

In this section we show how dense motion estimation can be combined with the trajectory subspace constraints described in Sect. 3. In order to estimate the 2D trajectories of all the points, or equivalently simultaneously register all the frames with the reference frame $n_0,$ we propose the following energy:

6 $$\begin{aligned} E [ \varvec{u}(\varvec{x}; n), \, \varvec{L}(\varvec{x}) ] = \alpha E_{data} + \beta E_{link} + E_{reg}, \end{aligned}$$

where

7 $$\begin{aligned} E_{data} = \int _\Omega \sum _{n=1}^F \left| I\left( \varvec{x}+\varvec{u}(\varvec{x};n) \,\,;\,\, n \right) - I(\varvec{x}; n_0) \right| \, \mathrm{d}\varvec{x}, \end{aligned}$$

8 $$\begin{aligned} E_{link} = \int _\Omega \sum _{n=1}^F \left|\varvec{u}(\varvec{x}; n) - \sum _{i=1}^R \varvec{q}_i(n) L_i(\varvec{x}) \right|^2 \mathrm{d}\varvec{x}, \end{aligned}$$

9 $$\begin{aligned} E_{reg} = \int _\Omega \sum _{i=1}^R \,\,g(\varvec{x}) \left|\nabla L_i(\varvec{x}) \right|_\epsilon \,\mathrm{d}\varvec{x}\,\, . \end{aligned}$$

We minimize this energy jointly with respect to the point trajectories $\varvec{u}(\varvec{x}; n)$ and their components on the trajectory subspace that are determined by the linear model coefficients $\varvec{L}(\varvec{x}).$ We also add the constraint that $\varvec{u}(\varvec{x}; n_0)=0,$ since this corresponds to the flow from the reference image frame to itself. The positive constants $\alpha$ and $\beta$ weigh the balance between the terms of the energy. Also, $|\cdot |_\epsilon$ in (9) denotes the Huber norm of a vector and $g(\varvec{x})$ is a space-varying weighting function (see Sect. 4 for more details).

Note that the functions $\varvec{u}(\varvec{x}; n)$ and $\varvec{L}(\varvec{x})$ determine two sets of trajectories that are relatively close to each other but not identical since the subspace constraint is imposed as a soft constraint.This improves the robustness of our method against overfitting to the image data in cases where the brightness constancy assumption fails. For this reason, we consider that the final output of our method are the trajectories $\varvec{\mathcal{U }}^{\prime }(\varvec{x}) = \mathrm{Q} \, \varvec{L}(\varvec{x})$ that lie on the trajectory subspace and are directly derived by the coefficients $\varvec{L}(\varvec{x}).$

### Description of the Energy

In this section we provide more details about the properties of the proposed energy (6).

The **first term** ($E_{data}$) is a data attachment term that uses the robust $\mathbf{L}^1$-norm and is a direct multi-frame extension of the brightness constancy term used by most optical flow methods, e.g. Zach et al. (2007). It is based on the assumption that the image brightness $I(\varvec{x}; n_0)$ at every pixel $\varvec{x}$ of the reference frame is preserved at its new location, $\varvec{x}+\varvec{u}(\varvec{x};n),$ in every frame of the sequence. The use of an $\mathbf{L}^1$-norm improves the robustness of the method since it allows deviations from this assumption, which might occur in real-world scenarios because of noise, illumination changes or occlusions of some points in some frames.

The **second term** ($E_{link}$) penalizes the difference between the two sets of trajectories $\varvec{u}(\varvec{x}; n)$ and $Q\varvec{L}(\varvec{x})$ and acts as a coupling (linking) term between them. This term serves as a soft constraint that the trajectories $\varvec{u}(\varvec{x}; n)$ should be relatively close to the subspace spanned by the basis $Q.$Concerning the weight $\beta ,$ the larger its value the more restrictive the subspace constraint becomes. Since the subspace of $Q$ is low-dimensional, this constraint operates also as a temporal regularization that is able to perform temporal filling-in in cases of occlusions or other distortions.

An equivalent interpretation is that this term is derived from the constraint that the error $\varvec{\varepsilon }(\varvec{x}; n)$ in (2) has a bounded $\mathbf{L}^2$ norm, i.e. $\int _\Omega \sum \limits _{n=1}^F \left|\varvec{\varepsilon }(\varvec{x}; n) \right|^2 \mathrm{d}\varvec{x}\le C,$ for some appropriate constant $C.$ Then $\beta$ corresponds to the Lagrange multiplier for this constraint.

The **third term** ($E_{reg}$) corresponds to the spatial regularization of the trajectory coefficients. This term penalizes spatial oscillations of each coefficient caused by image noise or other distortions but not strong discontinuities that are desirable in the borders of each object. In addition, this term allows to fill in textural information into flat regions from their neighbourhoods. Following Werlberger et al. (2009), Newcombe et al. (2011), we use the Huber norm over the gradient of each subspace coefficient $L_i(\varvec{x}),$ which is defined as:

10 $$\begin{aligned}&|\nabla L_i(\varvec{x}) |_\epsilon = H_\epsilon (|\nabla L_i(\varvec{x}) |^2), \text{ with: } \nonumber \\&H_\epsilon ( s^2 ) = \left\{ \begin{array}{ll} \frac{s^2}{2\epsilon } \,\, &{} \text{ if } s \le \epsilon \\ s- \frac{ \epsilon }{2} \,\, &{} \text{ otherwise } \end{array} \right. \end{aligned}$$

where $\epsilon$ is a relatively small constant. The Huber norm is a convex differentiable function that combines quadratic regularization in the interval $\left|\nabla L_i \right|\le \epsilon ,$ with Total Variation regularization outside the interval.For small gradient magnitudes the Huber norm offers smooth solutions, whereas for larger magnitudes the discontinuity preserving properties of Total Variation are maintained. Following Alvarez et al. (1999), Wedel et al. (2009), Newcombe et al. (2011), we also incorporate a space-varying weight $g(\varvec{x})$ that depends on the reference image as follows:

11 $$\begin{aligned} g(\varvec{x}) = \exp (-c_g |\nabla G_{\sigma _g}(\varvec{x}) *I(\varvec{x}; n_0) |^2) \end{aligned}$$

where $c_g$ is a constant and $\sigma _g$ is the standard deviation of the 2D Gaussian $G(\varvec{x})$ that convolves the reference image $I(\varvec{x}; n_0).$ This weight encourages discontinuities in flow to coincide with edges of the reference image by reducing the regularisation strength near those edges.Further discussion on our proposed regularization term $E_{reg}$ is provided in Sect. 4.

### Connections to Previous Work

Interestingly, our adopted strategy of estimating two sets of trajectories, $\varvec{u}(\varvec{x}; n)$ and $\mathrm{Q} \, \varvec{L}(\varvec{x}),$ resembles the techniques of quadratic relaxation and duplication of the optimization variable that have been previously used in the context of optical flow and depth map estimation (Zach et al. 2007; Pock et al. 2010; Stuehmer et al. 2010; Newcombe et al. 2011). Similarly, we benefit from the fact that the optimization problem can be decomposed into two parts, each of which is a convex energy^2^ that can be solved efficiently and in a globally optimal manner. However, our formulation offers an additional advantage: the spatial regularization step, which is the bottleneck in these optimization schemes, is computationally much more efficient since it is applied to the coefficients $\varvec{L}(\varvec{x})$ that normally have smaller dimensionality than the flow $\varvec{u}(\varvec{x}; n).$

Note that there is a degenerate case in which our proposed approach becomes equivalent to independently estimating the flow from the reference $I(\cdot ;n_0)$ to each frame $I(\cdot ;n)$ by applying $F-1$ times the ITV-$\mathbf{L}^1$ optical flow algorithm (Wedel et al. 2009). This degenerate case occurs when:

- The motion basis is set to $\mathrm{Q}=\mathrm{I}_{2F},$ where $\mathrm{I}_{2F}$ is the $2F \times 2F$ identity matrix, in which case $R=2F$; and
- $$c_g=0$$ and $$\epsilon =0.$$

When $c_g=0$ and $\epsilon =0,$ the terms $g(\varvec{x}) \left|\nabla L_i(\varvec{x}) \right|_\epsilon$ become equivalent to $\left|\nabla L_i(\varvec{x}) \right|$ and therefore our regularization term $E_{reg}$ is a summation of Total Variation terms. Furthermore, the choice $\mathrm{Q}=\mathrm I _{2F}$ converts the energy (6) into a summation of $F$ decoupled energy terms $J_n$:

12 $$\begin{aligned} J_n&= \alpha \int _\Omega \left| I\left( \varvec{x}+\varvec{u}(\varvec{x};n) \,\,;\,\, n \right) - I(\varvec{x}; n_0) \right| \, \mathrm{d}\varvec{x}\nonumber \\&+ \beta \int _\Omega \left|\varvec{u}(\varvec{x}; n) - \left[ \begin{array}{c} L_{2n-1}(\varvec{x})\\ L_{2n}(\varvec{x})\\ \end{array} \right] \right|^2 \mathrm{d}\varvec{x}\,\, \nonumber \\&+ \int _\Omega \sum _{i=2n-1}^{2n} \left|\nabla L_i(\varvec{x}) \right|\,\mathrm{d}\varvec{x}\end{aligned}$$

Each term $J_n$ corresponds to a specific frame $n$ and depends only on $\varvec{u}(\varvec{x};n)$ and the two coefficients $L_{2n-1}(\varvec{x})$ and $L_{2n}(\varvec{x}).$ These coefficients stacked together as a vector-valued function can be seen as the auxiliary variable of $\varvec{u}(\varvec{x};n)$ so the energy term $J_n$ is equivalent to the convex relaxation of the TV-$\mathbf{L}^1$ functional used in Wedel et al. (2009).

### Effective Trajectory Regularization

In this section we provide further intuition into our choice of multi-frame optical flow regularization $E_{reg}.$ The presentation of this section follows a constructive approach—we build our proposed regularizer from the simplest choice of regularization term in successive steps, each of which adds more complexity but improves its effectiveness. We start by revisiting common practices in the literature and conclude by proposing our novel *anisotropic trajectory regularization* term in the final step. Our goal is to regularize the multi-frame optical flow $\varvec{\mathcal{U }}^{\prime }(\varvec{x})$ that lies on the trajectory subspace. Note that $\varvec{\mathcal{U }}^{\prime }(\varvec{x})$ can be interpreted as a vector valued function with $2 \times F$ channels encoding the horizontal and vertical components of the optical flow at each frame as defined in equation (3).

*Step 1.* A simple choice would be to use homogeneous regularization of $\varvec{\mathcal{U }}^{\prime }(\varvec{x}),$ which is a straightforward multiframe generalization of the model of Horn and Schunck (1981):

13 $$\begin{aligned}&\int _\Omega \sum _{n=1}^F |\nabla u^{\prime }_1(\varvec{x}; n) |^2 + |\nabla u^{\prime }_2(\varvec{x}; n) |^2 \, \mathrm{d}\varvec{x}\nonumber \\&\quad = \int _\Omega \Vert D \, \varvec{\mathcal{U }}^{\prime }(\varvec{x}) \Vert ^2_F \, \mathrm{d}\varvec{x}\end{aligned}$$

where $\Vert \cdot \Vert _F$ denotes the Frobenius norm of a matrix and $D \, \varvec{\mathcal{U }}^{\prime }(\varvec{x})$ is the Jacobian of $\varvec{\mathcal{U }}^{\prime }(\varvec{x})$ (each row contains the gradient of the corresponding channel of $\varvec{\mathcal{U }}^{\prime }(\varvec{x})$). However, this regularizer leads to oversmoothing on the motion boundaries since the quadratic term excessively penalizes large magnitudes of the gradients of $\varvec{\mathcal{U }}^{\prime }(\varvec{x}),$ which correspond to motion discontinuities.

*Step 2.* A way to avoid this is by applying a *robust function* $\Psi$ that penalizes outliers of the gradient less severely than the quadratic penalizer:

14 $$\begin{aligned} \int _\Omega \Psi \left( \Vert D \, \varvec{\mathcal{U }}^{\prime }(\varvec{x}) \Vert ^2_F \right) \, \mathrm{d}\varvec{x}\end{aligned}$$

This choice is used in Nir et al. (2008) and when only two frames are taken into account it is equivalent to the regularizers used in Schnörr (1994), Weickert (1998), Brox and Malik (2011) (*isotropic flow-driven regularization* in the terminology of Weickert and Schnörr (2001a)). Some examples of the robust function $\Psi$ include the following:

- $\Psi (s^2) = s,$ in which case the regularizer is the *vectorial total variation* (Sapiro 1997) of the vector-valued function $\varvec{\mathcal{U }}^{\prime }(\varvec{x})$ that encodes the multi-frame optical flow.
- $\Psi (s^2) = H_\epsilon (s^2)$ or the Huber norm (10), which is the choice adopted in our approach.

The robust function $\Psi$ in (14) penalizes outliers of the norm $\Vert D \, \varvec{\mathcal{U }}^{\prime }(\varvec{x}) \Vert _F$ less strongly, therefore allows discontinuities to occur at $\varvec{\mathcal{U }}^{\prime }(\varvec{x}).$ However, such outliers correspond only to the points $\varvec{x}$ where all the channels of $\varvec{\mathcal{U }}^{\prime }(\varvec{x})$ display sharp discontinuities. If for example only few channels of $\varvec{\mathcal{U }}^{\prime }(\varvec{x})$ have a high gradient at a point $\varvec{x},$ then $\Vert D \, \varvec{\mathcal{U }}^{\prime }(\varvec{x}) \Vert _F$ is not treated as an outlier, since it is still low (because of the sum of squares over all channels that is involved in this norm). This regularizer is thus much less tolerant to motion boundaries that occur at individual channels.

*Step 3.*

The above problem can be addressed by applying the penalizer $\Psi$ independently to the squared norm of the gradient of each channel of $\varvec{\mathcal{U }}^{\prime }(\varvec{x})$:

15 $$\begin{aligned} \int _\Omega \sum _{n=1}^F \left\{ \Psi \left( |\nabla u^{\prime }_ 1(\varvec{x}; n) |^2 \right) + \Psi \left( |\nabla u^{\prime }_2(\varvec{x}; n) |^2 \right) \right\} \, \mathrm{d}\varvec{x}\end{aligned}$$

This is a direct multi-frame extension of the regularizer used in Deriche et al. (1995), Kumar et al. (1996), Aubert et al. (1999), Zach et al. (2007), Wedel et al. (2009) for which efficient numerical implementations exist (Zach et al. 2007; Wedel et al. 2009). In this way, each channel of $\varvec{\mathcal{U }}^{\prime }(\varvec{x})$ can have different boundaries. However, this regularizer is on the other extreme of the regularizer of Step 2: where substantial correlation between the different channels exists, it is ineffective since it allows correlated trajectories to have different boundaries.

In addition, in contrast to the regularizers proposed in previous steps, it is not rotation invariant (Weickert and Schnörr 2001a).

*Step 4.*

To avoid the aforementioned problems, we adopt our subspace model for the 2D trajectories $\varvec{\mathcal{U }}^{\prime }(\varvec{x})=\mathrm{Q} \varvec{L}(\varvec{x})$ and rewrite the norm $\Vert D \, \varvec{\mathcal{U }}^{\prime }(\varvec{x}) \Vert _F$ as a function of the coefficients:

16 $$\begin{aligned}&\Vert D \, \varvec{\mathcal{U }}^{\prime }(\varvec{x}) \Vert ^2_F = \left|\frac{\partial \varvec{\mathcal{U }}^{\prime }(\varvec{x})}{ \partial x_1} \right|^2 + \left|\frac{\partial \varvec{\mathcal{U }}^{\prime }(\varvec{x})}{\partial x_2} \right|^2 \nonumber \\&= \left|\mathrm{Q} \frac{\partial \varvec{L}(\varvec{x})}{\partial x_1} \right|^2 + \left|\mathrm{Q} \frac{ \partial \varvec{L}(\varvec{x})}{\partial x_2} \right|^2 = \sum _{i=1}^R \left|\nabla L_i(\varvec{x}) \right|^2, \end{aligned}$$

where we have used the property of orthonormality of the basis $\mathrm{Q}.$ Provided that the trajectory basis $\mathrm{Q}$ has been chosen appropriately, the coefficients $\varvec{L}(\varvec{x})$ are much less correlated than the channels of $\varvec{\mathcal{U }}^{\prime }(\varvec{x}).$ We conclude that it is more effective to apply the robust function $\Psi$ independently to the basis coefficients (instead of the flow fields) and we derive the regularizer:

17 $$\begin{aligned} \int _\Omega \sum _{i=1}^R \Psi \left( \left|\nabla L_i(\varvec{x}) \right|^2 \right) \, \mathrm{d}\varvec{x}\end{aligned}$$

Furthermore, this regularizer leads to a much more efficient implementation for two main reasons. First, the resultant regularization is applied to the coefficients $\varvec{L}(\varvec{x}),$ that typically have lower dimensionality than the flow $\varvec{\mathcal{U }}^{\prime }(\varvec{x}).$ Second, this regularization is decoupled for each coefficient and can thus be highly parallelized. Note that the regularizer (15) derived in Step 3 can be considered as a special case of the above regularizer when the $2F\times 2F$ identity matrix is chosen as the basis $\mathrm{Q}.$ However, in our work, we use two choices for $\mathrm{Q}$: DCT and PCA (derived from an initial flow). We now analyze each of these cases separately:

- When the basis matrix $\mathrm{Q}$ has been estimated by applying PCA to some trajectory samples, the correlation between the coefficients can be considered negligible. Furthermore, in this case we regain the desirable property of rotation invariance, since the proposed regularizer (17) is consistent with the general design principle of Weickert and Schnörr (2001a) for *rotationally invariant anisotropic regularizers*. According to that principle^3^, given an appropriate decomposition of $\Vert D \, \varvec{\mathcal{U }}^{\prime }(\varvec{x}) \Vert ^2_F = \sum _j \rho _j$ where $\rho _j$ are rotationally invariant expressions, one should use the regularizer $\int _\Omega \sum _j \Psi (\rho _j),$ which is rotationally invariant and anisotropic. In our case, the expressions $\rho _j$ correspond to the coefficients $L_i(\varvec{x}),$ which are indeed rotation invariant: If we assume that a rotation of the input frames causes the same rotation to be applied to the trajectory samples, then the basis trajectories will be equally rotated. Therefore, the coefficients $L_i(\varvec{x})$ of a specific reference image point 4 will remain invariant and the corresponding trajectory $\varvec{\mathcal{U }}^{\prime }(\varvec{x})$ will simply be rotated.
- In the case of the DCT basis, the above properties do not hold. However, the regularizer (17) with a DCT basis is much more effective than the regularizer (15), since the DCT frequency components of a trajectory are typically less correlated than its actual coordinates. This is due to the fact that when the actual motions of the image points are compositions of different physical motions, these motions are expected to be much more localized in the frequency domain rather than in the time domain.

*Step 5.*

Finally, it is reasonable to assume that the boundaries of all the motion components tend to be a subset of the edges on the reference image. Following Alvarez et al. (1999), Wedel et al. (2009), Newcombe et al. (2011), in order to prevent any smoothing along the motion boundaries our final regularizer $E_{reg}$ is weighted by a space-varying function $g(\varvec{x})$ that depends on the reference image as described in (11).

In our extensive experiments, we have empirically evaluated that the introduction of such a weighting improves the accuracy of the multiframe optical flow. This is in accordance with the experimental evidence reported in Wedel et al. (2009) for the classical optical flow.

## Optimization of the Proposed Energy

In order to minimize the energy (6), we follow a coarse-to-fine technique with multiple warping iterations (Brox et al. 2004). In every warping iteration, we use an initialization $\varvec{u}_0(\varvec{x};n)$ that comes from the previous iteration. We approximate the data term (7) by linearizing the image $I(\cdot ;n)$ around $\varvec{x}+\varvec{u}_0(\varvec{x}; n).$ After this approximation, the energy (6) becomes convex.

Following Zach et al. (2007), we implement the optimization of the energy (6) using an alternating approach. We decouple the data and regularization terms to decompose the optimization problem into two, each of which can be more easily solved. In this section we show how to adapt the method of Zach et al. (2007) to our problem, to take advantage of its computational efficiency and apply it to multi-frame subspace-constrained optical flow. The key difference to Zach et al. (2007) is that we do not solve for pairwise optical flow but instead we optimize over all the frames of the sequence while imposing the trajectory subspace constraint as a soft constraint.

We apply an alternating optimization, updating either $\varvec{u}(\varvec{x}; n)$ or $\varvec{L}(\varvec{x})$ in every iteration, as follows:

- Repeat until convergence: **Minimization Step 1:** For $\varvec{u}(\varvec{x}; n)$ fixed, update $\varvec{L}(\varvec{x})$ by minimizing $E[ \varvec{u}(\varvec{x}; n), \varvec{L}(\varvec{x})] \;$w.r.t.$\; \varvec{L}(\varvec{x}).$ **Minimization Step 2:** For $\varvec{L}(\varvec{x})$ fixed, update $\varvec{u}(\varvec{x}; n)$ by minimizing $E[ \varvec{u}(\varvec{x}; n), \varvec{L}(\varvec{x})] \;$w.r.t.$\; \varvec{u}(\varvec{x}; n).$

Convergence is declared if the relative update of $\varvec{L}(\varvec{x})$ and $\varvec{u}(\varvec{x}; n)$ is negligible according to some appropriate distance threshold. Since at every step the value of the energy does not increase and this value is bounded below by its global minimum, the above alternation is guaranteed to converge to a global minimum point.

### Minimization Step 1

Since in this step we keep $\varvec{u}(\varvec{x}; n)$ fixed, we observe that only the last two terms, $E_{link}$ and $E_{reg},$ of the energy (6) depend on $\varvec{L}(\varvec{x}).$ Therefore we must minimize $\beta E_{link}+E_{reg}$ with respect to $\varvec{L}(\varvec{x}).$ Using the matrix notation defined in (4), we can write the term $E_{link}$ as:

18 $$\begin{aligned} \sum _{n=1}^F |\varvec{u}(\varvec{x};\! n) \!-\! \sum _{i=1}^R \varvec{q}_i(n) L_i(\varvec{x}) |^2 = |\varvec{\mathcal{U }}(\varvec{x}) \!-\! \mathrm{Q} \varvec{L}(\varvec{x}) |^2 \end{aligned}$$

Let $\mathrm{Q}^\bot$ be an $2F \times (2F-R)$ matrix whose columns form an orthonormal basis of the orthogonal complement of the trajectory subspace $\mathcal{S }_Q.$ Then the block matrix $[\mathrm{Q} \, \, \mathrm{Q}^\bot ]$ is an orthonormal $2F \times 2F$ matrix, which means that its columns form a basis of $\mathbb{R }^{2F}.$ Consequently, $\varvec{\mathcal{U }}(\varvec{x})$ can be decomposed into two orthonormal vectors as

19 $$\begin{aligned} \varvec{\mathcal{U }}(\varvec{x}) = \mathrm{Q} \, \varvec{M}(\varvec{x}) + \mathrm{Q}^\bot \, \varvec{M}_{out}(\varvec{x}) \end{aligned}$$

where

20 $$\begin{aligned} \varvec{M}(\varvec{x}) \triangleq \mathrm{Q}^T \varvec{\mathcal{U }}(\varvec{x})\quad \text{ and } \quad \varvec{M}_{out}(\varvec{x}) \triangleq (\mathrm{Q}^\bot )^T \varvec{\mathcal{U }}(\varvec{x}) \end{aligned}$$

are the coefficients that define the projections of $\varvec{\mathcal{U }}(\varvec{x})$ onto the trajectory subspace $\mathcal{S }_Q$ and its orthogonal complement. Equation (18) can now be further simplified:

21 $$\begin{aligned}&|\varvec{\mathcal{U }}(\varvec{x}) - \mathrm{Q} \, \varvec{L}(\varvec{x}) |^2 \nonumber \\&\quad = \left|\mathrm{Q}^\bot \, \varvec{M}_{out}(\varvec{x}) \!+\! \mathrm{Q} \left( \varvec{M}(\varvec{x}) - \varvec{L}(\varvec{x}) \right) \right|^2\nonumber \\&\quad = \left|\varvec{M}_{out}(\varvec{x}) \right|^2 \!+\! \left|\varvec{M}(\varvec{x}) - \varvec{L}(\varvec{x}) \right|^2, \end{aligned}$$

due to the orthonormality of the columns of $\mathrm{Q}$ and $\mathrm{Q}^\bot$ (which makes the corresponding transforms isometric) and Pythagoras’ theorem. The component $\left| \varvec{M}_{out}(\varvec{x}) \right| ^2$ is constant with respect to $\varvec{L}(\varvec{x})$; therefore it can be ignored from the current minimization. In other words, with $\varvec{\mathcal{U }}$ being fixed and $\mathrm{Q} \, \varvec{L}$ lying on the linear subspace $\mathcal{S }_Q,$ penalizing the distance between $\mathrm{Q} \, \varvec{L}$ and $\varvec{\mathcal{U }}$ is equivalent to penalizing the distance between $\mathrm{Q} \, \varvec{L}$ and the projection of $\varvec{\mathcal{U }}$ onto $\mathcal{S }_Q.$

Thus, the minimization of Step 1 is equivalent to the minimization of:

22 $$\begin{aligned}&\beta \int _\Omega |\varvec{M}(\varvec{x}) - \varvec{L}(\varvec{x}) |^2 \mathrm{d}\varvec{x}+\int _\Omega \sum _{i=1}^R g(\varvec{x}) |\nabla L_i(\varvec{x}) |_\epsilon \mathrm{d}\varvec{x}\nonumber \\&\quad \!=\! \sum _{i=1}^R \int _\Omega \, g(\varvec{x}) \left|\nabla L_i(\varvec{x}) \right|_ \epsilon \! +\! \beta ( M_i(\varvec{x}) \!-\!L_i(\varvec{x}) ) ^2 \mathrm{d}\varvec{x}\nonumber \\ \end{aligned}$$

where $M_i(\varvec{x})$ is the $i$-th coordinate of $\varvec{M}(\varvec{x}).$ We have finally obtained a new form of the energy that allows the trajectory model coefficients $L_i(\varvec{x})$ to be decoupled. The minimization of each term in the above sum can be done independently and corresponds to a small modification of the TV-$\mathbf{L}^2$ Rudin-Osher-Fatemi (ROF) model (Rudin et al. 1992) applied to each coefficient $L_i(\varvec{x})$: This modification consists of incorporating an edge weighting $g(\varvec{x})$ and replacing the $\mathbf{L}^2$ norm $\left|\nabla L_i(\varvec{x}) \right|$ with the Huber norm $\left|\nabla L_i(\varvec{x}) \right|_\epsilon .$ This modified ROF model has been recently studied in Newcombe et al. (2011) for the problem of depth estimation.The optimum $L_i(\varvec{x})$ is actually a regularized version of $M_i(\varvec{x})$ and the extent of this regularization increases as the weight $\beta$ decreases.

The benefits of the computational efficiency of the above procedure are twofold. First, these independent minimizations can be parallelized. Second, several efficient algorithms exist to implement such regularization models. Appendix A describes the actual algorithm we used for the optimization of this energy, which is related to the method proposed in Newcombe et al. (2011).

### Minimization Step 2

Keeping $\varvec{L}(\varvec{x})$ fixed, we observe that only the first two terms of the energy (6), $E_{data}$ and $E_{link},$ depend on $\varvec{u}(\varvec{x}; n)$ and therefore we have to minimize with respect to $\varvec{u}(\varvec{x}; n)$ the following:

23 $$\begin{aligned}&\alpha E_{data} + \beta E_{link} = \nonumber \\&\quad \int _\Omega \sum _{n=1}^F \biggl \{ \alpha \left| I\left( \varvec{x}+\varvec{u}(\varvec{x};n) \,\,;\,\, n \right) - I(\varvec{x}; n_0) \right| \nonumber \\&\qquad \qquad \quad + \beta \left|\varvec{u}(\varvec{x}; n) - \varvec{u}^{\prime } \right|^2 \biggr \} \,\mathrm{d}\varvec{x}, \end{aligned}$$

where $\varvec{u}^{\prime }=\sum _{i=1}^R \varvec{q}_i(n) L_i(\varvec{x}).$ This cost depends only on the value of $\varvec{u}$ on the specific point $\varvec{x}$ and the discrete time $n$ (and not on the derivatives of $\varvec{u}$). Therefore the variational minimization of Step 2 is equivalent to the minimization of a bivariate function of $\varvec{u}$ for every spatiotemporal point $(\varvec{x};n)$ independently.

We implement this point-wise minimization by applying the technique proposed in Zach et al. (2007) to every frame. More precisely, for every frame $n$ and point $\varvec{x}$ the image $I(\cdot ;n)$ is linearized around $\varvec{x}+\varvec{u}_0(\varvec{x}; n),$ where $\varvec{u}_0(\varvec{x}; n)$ are the initializations of the trajectories $\varvec{u}(\varvec{x};n).$ The function to be minimized at every point will then have the simple form of a summation of a quadratic term with the absolute value of a linear term. The minimum can be easily found analytically using the thresholding scheme reported in Zach et al. (2007).

## Derivation of the Trajectory Basis

Concerning the choice of 2D trajectory basis $\mathrm{Q},$ we consider orthonormal bases as it simplifies the analysis and calculations in our method (see Sect. 4). Of course this assumption is not restrictive, since for any basis an orthonormal one can be found that will span the same subspace. We now describe several effective choices of trajectory basis that we have used in our formulation.

Predefined bases for single-valued discrete-time signals with $F$ samples can be used to model separately each coordinate of the 2D trajectories. Assuming that the rank $R$ is an even number, this single-valued basis should have $R/2$ elements $w_1(n),\ldots ,w_{R/2}(n)$ and the trajectory basis would be given by:

24 $$\begin{aligned} \mathbf{q}_{i}(n) = {\left\{ \begin{array}{ll} {[{w}_{i}(n), 0]}^{T}, &{} \text{ if } i=1,\ldots ,\frac{R}{2}\\ {[0, {w}_{i-R/2}(n)]}^{T}, &{} \text{ if } i=\frac{R}{2}+1,\ldots ,R \end{array}\right. } \end{aligned}$$

Provided that the object moves and deforms smoothly, effective choices for the basis $\{w_i(n)\}$ are (*i*) the first $\frac{R}{2}$ low-frequency basis elements of the 1D Discrete Cosine Transform (DCT) or (*ii*) a sampling of the basis elements of the Uniform Cubic B-Splines of rank $R/2$ over the sequence’s time window, followed by orthonormalization of the yielded basis. The obvious advantage of using a predefined basis is that it does not need to be estimated in advance.

An alternative is to estimate the basis by applying Principal Component Analysis (PCA) to some sample trajectories. Provided that it is possible to estimate a set of sample trajectories that adequately represent the trajectories of the points over the whole object, the choice of the PCA basis is optimum for the linear model of a given rank $R,$ in terms of representational power. In this work we consider two possibilities.

(i) The sample trajectories could come from an initial estimate of optical flow. We have found that the flow obtained using the DCT basis provides a very good initial flow on which we then apply PCA to obtain an optimized basis.
(ii) Alternatively, the sample trajectories could be a small subset of *reliable* point tracks, which we consider to be those where the texture of the image is strong in both spatial directions and can be selected using Shi and Tomasi’s criterion (Shi and Tomasi 1994). However, this option is not resilient to outliers.

In practice, in our experimental evaluation section we show that the multi-frame optical flow obtained with the optimized PCA basis proposed in (i) provides the best results. It has the added advantage that, since we initialize the flow from our algorithm using the DCT basis, which is predefined and needs not be estimated, the entire process is automated and less affected by outliers.

## Generalization to Sequences of Vector-Valued Images

The algorithm we have described so far assumes that the images in the sequence are grayscale. In this section we develop a generalization of our approach to the case of sequences of vector-valued images. We propose an optimization scheme that is based on the dualization of the data term of the energy.

The use of vector-valued images can significantly improve the accuracy of the estimated optical flow for various reasons. First of all, the vector-valued images can incorporate all the color channels of an image. The color cue in a video offers important additional information and resolves ambiguities that are present in the grayscale images. Furthermore, this generalization offers the potential for incorporating other powerful image cues as additional channels. For instance, the spatial derivatives of the color channels can be added to impose the gradient constancy assumption (Uras et al. 1988; Brox et al. 2004; Papenberg et al. 2006; Brox and Malik 2011) or even more complex features such as SIFT (Liu et al. 2011) features or others derived using a Field-of-Experts formulation (Sun et al. 2008), which can improve the robustness against illumination changes of the scene. Note that in our experimental evaluation we have only incorporated the color channels. To cope with illumination changes we have used structure-texture decomposition as a preprocessing step, which is an alternative way to gain robustness (Wedel et al. 2009).

### Proposed Dual Formulation 

Let us assume that the video frames that are used in our data term are vector-valued images with $N_c$ channels:

25 $$\begin{aligned} \varvec{I}(\varvec{x};n):\Omega \times \{1,\ldots ,F\} \rightarrow \mathbb{R }^{N_c} \end{aligned}$$

To cope with this more general case, we only have to modify two elements of the formulation of our energy: (i) the data term $E_{data}$ of the proposed energy (6) and (ii) the edge-weighting function of the regularization term $g(\varvec{x})$ described in (11) that depends on the reference image.

The original definition of the function $g(\varvec{x})$ is based on the term $|\nabla G_{\sigma _g}(\varvec{x}) *I(\varvec{x}; n_0) |^2$ used as a simple edge-strength predictor. For vector-valued images, we use a common and natural extension of this predictor (Blomgren and Chan 1998; Tschumperlé and Deriche 2005) by adding the contributions of the different image channels. We thus generalize the edge-weighting function as follows:

26 $$\begin{aligned} g(\varvec{x}) = \exp \left( -c_g \sum _{i=1}^{N_c} |\nabla G_{\sigma _g}(\varvec{x}) *I_i(\varvec{x}; n_0) |^2 \right) \end{aligned}$$

Concerning the data term $E_{data},$ we also make a further generalization by applying a generic robust function 5 $\Phi$ to the image differences:

27 $$\begin{aligned} \Phi : \mathbb{R }^{N_c} \rightarrow \mathbb{R }. \end{aligned}$$

Our generalized data term becomes:

28 $$\begin{aligned} E_{data} = \int _\Omega \sum _{n=1}^F \Phi \left( \varvec{I}\left( \varvec{x}+\varvec{u}\,\,;\,\, n \right) - \varvec{I}(\varvec{x}; n_0) \right) \mathrm{d}\varvec{x}\end{aligned}$$

Since only the data term is affected by the extension to vector-valued images, the optimization of our proposed energy (6) only requires a modification of the minimization of $\alpha E_{data} + \beta E_{link}$ with respect to $\varvec{u}(\varvec{x}; n)$ (Step 2 in Sect. 5). Similarly to the case of grayscale images, this minimization is independent for every spatio-temporal point $(\varvec{x};n).$ But the point-wise energy that must be minimized with respect to $\varvec{u}$ is now the following:

$$\begin{aligned} E^{aux}(\varvec{u}) = \alpha \Phi \left( \varvec{I}\left( \varvec{x}+\varvec{u}\,\,;\,\, n \right) - \varvec{I}(\varvec{x}; n_0) \right) + \beta \left|\varvec{u}- \varvec{u}^{\prime } \right|^2 \end{aligned}$$

For every point $\varvec{x}$ in every frame $n$ each channel of $\varvec{I}(\cdot ;n)$ is linearized around $\varvec{x}+\varvec{u}_0(\varvec{x}; n),$ where $\varvec{u}_0(\varvec{x}; n)$ are the initializations of the trajectories $\varvec{u}(\varvec{x};n).$ With this approximation, $E^{aux}$ can be written as:

29 $$\begin{aligned} E^{aux}(\varvec{u}) = \alpha \Phi \left( {\varvec{A}\varvec{u}+ \varvec{b}} \right) + \beta \left|\varvec{u}- \varvec{u}^{\prime } \right|^2 \end{aligned}$$

where $\varvec{b}= I(\varvec{x}+\varvec{u}_0;n) - I(\varvec{x};n_0) - \varvec{A}\varvec{u}_0$ and $\varvec{A}= \frac{\partial \varvec{I}(\varvec{x}+ \varvec{u}_0 ; n)}{\partial \varvec{x}}$ is the $N_c\times 2$ (spatial) Jacobian of the $n$-th frame $\varvec{I}(\cdot ;n),$ evaluated at $\varvec{x}+ \varvec{u}_0.$

Assuming that the function $\Phi$ is proper convex and lower semi-continuous, we dualise it by using its convex bi-conjugate (Rockafellar 1997; Chambolle and Pock 2011):

30 $$\begin{aligned} \Phi (\varvec{s}) = \sup _\mathcal{I } \{\langle \varvec{s},\mathcal{I } \rangle - \Phi ^{*}(\mathcal{I }) \} \end{aligned}$$

where, $\Phi ^{*}(\mathcal{I })$ is the Legendre-Fenchel transform of $\Phi (s)$ and $\mathcal{I }$ is the dual variable to $s.$ We can now rewrite the energy $E^{aux}$ (29) as:

31 $$\begin{aligned} E^{aux}(\varvec{u})&= \alpha \max _{\varvec{\mathcal{I }}} \{ \langle \varvec{A}\varvec{u}+ \varvec{b}, \varvec{\mathcal{I }}\rangle - \Phi ^{*}(\varvec{\mathcal{I }}) \} \nonumber \\&+ \beta \left|\varvec{u}- \varvec{u}^{\prime } \right|^2 \end{aligned}$$

Based on the above expression, we propose to minimise $E^{aux}$ by solving the following saddle point problem:

32 $$\begin{aligned} \min \limits _{\varvec{u}} \max \limits _{\varvec{\mathcal{I }}} \,\, E^{sp}(\varvec{u},\varvec{\mathcal{I }}), \end{aligned}$$

where

33 $$\begin{aligned} E^{sp}(\varvec{u},\varvec{\mathcal{I }})&\triangleq \alpha \left( \langle \varvec{A}\varvec{u}+ \varvec{b}, \varvec{\mathcal{I }}\rangle - \Phi ^{*}(\varvec{\mathcal{I }}) \right) \nonumber \\&+\, \beta \left|\varvec{u}- \varvec{u}^{\prime } \right|^2 \end{aligned}$$

Given a specific choice for the robust function $\Phi ,$ one can derive efficient algorithms to solve the saddle point problem (32), using a similar framework as in Esser et al. (2010), Chambolle and Pock (2011), Pock and Chambolle (2011). In Appendix B we provide such algorithms for two special cases of $\Phi$ of particular interest:

- $\Phi (\varvec{v})=|\varvec{v}|,$ which leads to $\mathbf{L}^1$- norm of the image differences in $E_{data}$ (28). This is the choice that we use in our experiments on colour images.
- $\Phi (\varvec{v})=H_\epsilon (|\varvec{v}|^2),$ which corresponds to the Huber norm (10).

Note that Rakêt et al. (2011) recently proposed an extension of the TV-$\mathbf{L}^1$ algorithm for vector-valued images. Their method corresponds to the choice $\Phi (\varvec{v})=|\varvec{v}|$ and uses a step of projection onto an elliptic ball. The formulation that we propose in this section can be seen as an alternative to the aforementioned work. The advantage of our approach is that it allows the use of more general robust functions $\Phi .$

## Implementation Details

In this section we provide details about the implementation of the numerical optimization schemes for our grayscale and vector-valued multi-frame subspace optical flow algorithms.

We used a similar numerical optimisation scheme and preprocessing of images^6^ to the one proposed in Wedel et al. (2009) to minimise the energy (6), i.e. we use the structure-texture decomposition to make our input robust to illumination artifacts due to shadows and shading reflections. We also used blended versions of the image gradients and a median filter to reject flow outliers. Concerning the choice of parameters, the default values proposed in Wedel et al. (2009) for the ITV-$\mathbf{L}^1$ algorithm were found to give the best results for ITV-$\mathbf{L}^1$ and our method on the benchmark sequence (5 warp iterations, 20 alternation iterations and the weights $\alpha$ and $\beta$ were set to 30 and 2). The same settings were used in all our experiments on real sequences. Note that when we ran the colour version of our algorithm we downweighed the value of $\alpha$ by a factor of $1 \over \sqrt{3}$ to account for the three colour channels. Regarding the parameters of the space varying weight of the regularization term $g(\varvec{x})$ defined in (11), we used the following values: $\sigma _g= 1$ pixel, $c_g = 0.8$ and $\epsilon = 0.1.$

Since our algorithm can be efficiently parallelized on standard graphics hardware we have developed a GPU implementation using the CUDA framework. We run our algorithm on an NVIDIA GTX-580 GPU card hosted on a dual-core CPU. We obtain an average speedup of $\times 50$ with respect to our CPU Matlab implementation which runs on a 4 quad-core server with 192Gb of memory.

## Reparameterization of the Optical Flow: Hard Subspace Constraint

In the special case where the error $\varvec{\varepsilon }(\varvec{x}; n)$ in (2) is close to zero everywhere in the image, or equivalently when $\beta \rightarrow \infty$ in (6), our soft constraint becomes a hard constraint and the optical flow $\varvec{u}(\varvec{x};n)$ can be reparameterized as:

34 $$\begin{aligned} \sum \limits _{i=1}^R \varvec{q}_i(n) L_i(\varvec{x}) \end{aligned}$$

where the coefficients of the motion basis $L_i(\varvec{x})$ are the unknown variables. In this case the energy for vector valued images with $N_c$ channels can be rewritten as:

35 $$\begin{aligned} E_{h}&= \int _\Omega \sum _{n=1}^F \left| \varvec{I}\left( \varvec{x}+ Q_n \varvec{L}(\varvec{x}) \,\,;\,\, n \right) - \varvec{I}(\varvec{x}; n_0) \right| \, \mathrm{d}\varvec{x}\nonumber \\&+\int _\Omega \sum _{i=1}^R \,\,g(\varvec{x}) \left|\nabla L_i(\varvec{x}) \right|_\epsilon \, \mathrm{d}\varvec{x}\end{aligned}$$

where $Q_n$ is the $2 \times R$ matrix $\left[ \varvec{q}_1(n) \cdots \varvec{q}_R(n) \right] ,$ i.e. two rows of the basis matrix ${Q}$ which correspond to frame $n.$ Appendix C describes a primal-dual optimization algorithm to minimize this energy obtained via reparameterization of the flow.

A valid question at this point would be: how does this hard subspace constraint compare with respect to our proposed soft constraint? In Sect. 3 we argued that a soft constraint would provide increased robustness. For this reason, in Sect. 10 we have conducted a thorough experimental comparison between the two approaches which in fact reveals that it is indeed beneficial to allow deviations from the subspace constraint. Our robust soft constraint consistently outperforms imposing a hard constraint via reparameterization of the optical flow.

## Experimental Results

In this section we evaluate our method and compare its performance with state of the art optical flow (Brox and Malik 2011; Zach et al. 2007) and image registration (Pizarro and Bartoli 2010) algorithms. We show quantitative comparative results on our new benchmark ground truth optical flow dataset and qualitative results on real-world sequences^7^.

Furthermore, we analyse the sensitivity of our algorithm to some of its parameters, such as the choice of trajectory basis and regularization weight. Since our algorithm computes multi-frame optical flow and incorporates an implicit temporal regularization term, it would have been natural to compare its performance with a spatiotemporal optical flow formulation such as Weickert and Schnörr (2001b). However, due to the lack of publicly available implementations we chose to compare with LDOF (Large Displacement Optical Flow) (Brox and Malik 2011), one of the best performing optical flow algorithms, that can deal with large displacements by integrating rich feature descriptors into a variational optic flow approach to compute dense flow. We also compare against the duality-based ITV-$\mathbf{L}^1$ (Improved TV-$\mathbf{L}^1$) algorithm (Wedel et al. 2009), which we use as a baseline since our method can be seen as its generalization to the case of multi-frame non-rigid optical flow via robust trajectory subspace constraints (see Sect. 4). In both cases, we register each frame in the sequence independently with the reference frame. We also compare with Pizarro and Bartoli’s state of the art keypoint-based non-rigid registration algorithm (Pizarro and Bartoli 2010).

Note that all these algorithms can only be used on grayscale images.

### Construction of a Ground Truth Benchmark Dataset

**Fig. 4:**
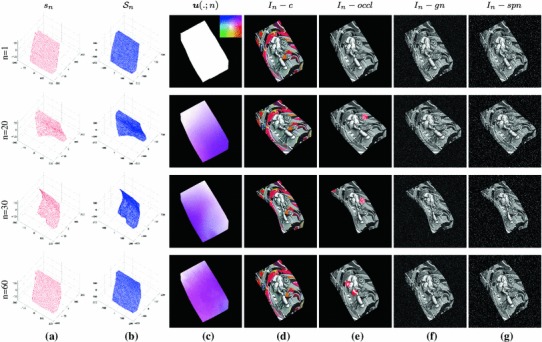
Rendering process for ground truth optical flow sequence of a non-rigid object for different images in each row. (**a**) Sparse surface $s_n$ representing MOCAP data (White et al. 2007), (**b**) Dense surfaces ${\mathcal{S }}_n$ constructed using thin plate spline interpolation, (**c**) Ground truth optical flow $\varvec{u}(\varvec{x};n)$ visualized with the color coding that is shown at $n=1,$ (**(d)**) Color sequence $I_n-c$ rendered from ${\mathcal{S }}_n$ using texture mapping of a graffiti image, (**e**) Grayscale version $I_n-occl$ of the same sequence with superimposed red disks indicate regions where intensities are replaced by black in the case of synthetic occlusions, (**f**) Grayscale sequence $I_n-gn$ with synthetic gaussian noise, (**g**) Grayscale sequence $I_n-spn$ with synthetic salt and pepper noise (Color figure online)

For the purpose of quantitative evaluation of multi-frame non-rigid optical flow we have generated a new benchmark sequence with ground truth optical flow data. To the best of our knowledge, this is one of the first attempts to generate a long image sequence of a deformable object with dense ground truth 2D trajectories. We use sparse motion capture (MOCAP) data from White et al. (2007) to capture the real deformations of a waving flag in 3D. This sparse data is interpolated to create a continuous dense 3D surface using the motion capture markers as the control points for smooth Spline interpolation. Figure 4 shows four frames of the (a) sparse and (b) dense interpolated 3D flag surface. This dense 3D surface is then projected synthetically onto the image plane using an orthographic camera. We use texture mapping to associate some texture to the surface while rendering 60 frames of size 500$\times$500 pixels. We provide both grayscale and colour sequences.The advantage of this new sequence is that, since it is based on MOCAP data, it captures the complex natural deformations of a real non-rigid object while allowing us to have access to dense ground truth optical flow. We have also used three degraded versions of the original rendered sequences by adding (i) Gaussian noise, of standard deviation 0.2 relative to the range of image intensities, (ii) salt & pepper (S&P) noise of density 10% and (iii) synthetic occlusions generated by superimposing some black circles of radius 20 pixels moving in linear orbits. Figure 4 shows four frames of the original colour sequence, the ground truth optical flow and the equivalent frames of the grayscale sequence with: synthetic occlusions, Gaussian noise and salt & pepper noise.

### Quantitative Results on Benchmark Sequence

**Fig. 5:**
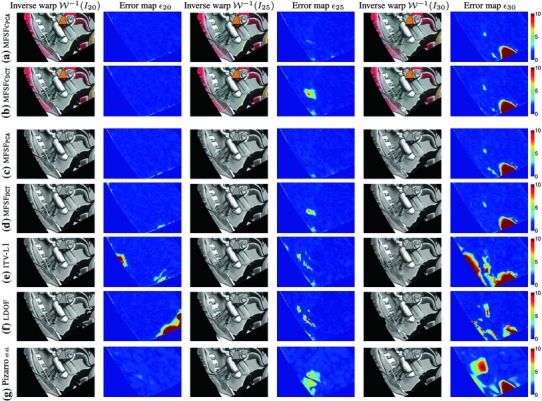
Inverse warps $\mathcal{W }^{-1}(I_i)$ and error maps $\epsilon _i(\varvec{x})$ for frames ($i=20, 25, 30$) of the original benchmark sequence. Each row shows results for different methods. (**a–b**) Multi-frame subspace flow on color images: (**a**) mfsf *c* $_\mathtt{PCA}$, (**b**) mfsf *c* $_\mathtt{DCT}$. (**c–d**) Multi-frame subspace flow on grayscale images: (**c**) mfsf $_\mathtt{PCA}$, (**d**) mfsf $_\mathtt{DCT}$. Against (**e**) itv-l1, Wedel et al. (2009). (**f**) LDOF Brox and Malik (2011), (**g**) Pizarro and Bartoli (2010)

**Fig. 6:**
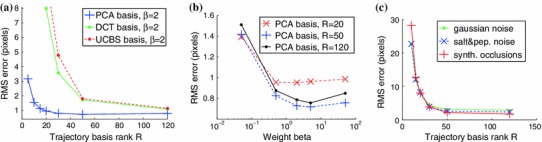
(**a**) RMS flow error vs increasing values of the rank of the different trajectory bases (PCA, DCT, UCBS). The graph shows that the PCA motion basis provides best results and that our algorithm does not overfit when the rank of the basis is overestimated. (**b**) RMS flow error vs increasing values of the weight of the subspace constraint $\beta .$ (**c**) RMS flow error for increasing value of the rank of the PCA basis on the different variants of the benchmark sequence (occlusions, Gaussian noise, salt & pepper noise). All experiments are for our grayscale multi-frame subspace flow algorithm $\textsc {mfsf}$

**Fig. 7:**
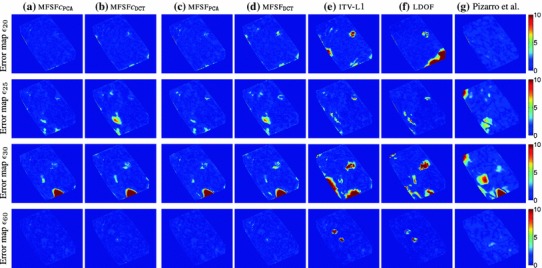
Flow error maps $\epsilon _i(\varvec{x})$ on the benchmark sequence with synthetic occlusions for frames ($i=20, 25, 30, 60$). Each column shows results for different methods and errors are displayed as heatmaps. (**a–b**) Multi-frame subspace flow on color images: (**a**) mfsf *c* $_\mathtt{PCA}$, (**b**) mfsf *c* $_\mathtt{DCT}$. (**c–d**) Multi-frame subspace flow on grayscale images: (**c**) mfsf $_\mathtt{PCA}$, (**d**) mfsf $_\mathtt{DCT}$. (**e**) itv- **L** $^{{1}}$ Wedel et al. (2009). (**f**) ldof Brox and Malik (2011) (**g**) Pizarro and Bartoli (2010). It is easy to see from the error maps for frames $20$ or $25$ that the colour versions of our algorithm (**a**) mfsf *c* $_\mathtt{PCA}$ and (**b**) mfsf $_\mathtt{DCT}$ improve substantially on their grayscale counterparts (**c**) mfsf $_\mathtt{PCA}$ and (**d**) mfsf $_\mathtt{DCT}$

We tested our *Multi-Frame Subspace Flow* algorithm for grayscale (mfsf) and colour images (mfsf c) using the three different proposed motion basis: PCA, DCT and Cubic B-Spline (Figs. 5, 6). In Table 1, we provide a quantitative comparison of the performance of the different versions of our algorithm, against the state of the art methods listed above, using the four different versions of the rendered flag sequence as input. We report the *root mean square* (RMS) of the endpoint error, i.e. the amplitude of the difference between the ground truth and estimated flow $\varvec{u}(\varvec{x};n).$ These measures are computed over all the frames and for all the foreground pixels. Note that the results obtained with the Spline basis were omitted since they were almost equivalent to those obtained with the DCT basis, as Fig. 7a reveals.

**Table 1:** RMS endpoint errors in pixels on the benchmark sequences of our proposed method for colour (mfsf *c*) and grayscale (mfsf) images using different motion basis (PCA, DCT and I $_{2F}$)

Image type	Method	Version of input sequence:			
		Original	Occlusions	Gauss. noise	S&P noise
Color	$\textsc {mfsf} c_{\mathtt{PCA}}$	**0.69**	**0.80**	**1.25**	**1.01**
	$\textsc {mfsf} c_{\mathtt{DCT}}$	0.80	1.00	1.52	1.17
	$\textsc {mfsf}_{\mathtt{PCA}}$	**0.75**	**0.85**	**1.52**	**1.18**
	$\textsc {mfsf}_{\mathtt{DCT}}$	0.89	1.12	1.84	1.38
Grayscale	$\textsc {mfsf}_{\mathtt{I_{2F}}}$	1.13	1.43	1.83	1.60
	ITV-$\mathbf{L}^1$ (Wedel et al. 2009)	1.43	1.89	2.61	2.34
	LDOF (Brox and Malik 2011)	1.71	2.01	4.35	5.05
	Pizarro and Bartoli (2010)	1.24	1.27	1.94	1.79

We compare the different versions of our grayscale algorithm (mfsf) against state of the art optical flow (ITV-$\mathbf{L}^1$ (Wedel et al. 2009), LDOF (Brox and Malik 2011)) and non-rigid registration (Pizarro and Bartoli 2010) methods

Numbers in bold highlight best performing color/grayscale algorithm

First we compare the performance of our original algorithm for grayscale images (mfsf) with ITV-$\mathbf{L}^1$  (Wedel et al. 2009), LDOF Brox and Malik (2011) and Pizarro and Bartoli (2010), since these algorithms can only be used on grayscale images. We report results for our algorithm using the full rank ($R=2F$) DCT basis (mfsf $_\mathtt{DCT}$) and a full rank PCA basis (mfsf $_\mathtt{PCA}$). Note that the PCA basis was estimated using as input the flow obtained after running our algorithm with the DCT basis (mfsf $_\mathtt{DCT}$). We also ran our algorithm using the identity matrix as the basis (mfsf $_{\mathtt{I}_{2F}}$) to show the degradation of the results when subspace constraints are not applied to compute the multi-frame optical flow.

Table 1 shows that our proposed algorithms (mfsf $_\mathtt{PCA}$) and (mfsf $_\mathtt{DCT}$) rank top amongst the grayscale algorithms, outperforming all other methods and yielding the lowest RMS errors on all the sequences: original, occlusions, Gaussian noise and salt & pepper noise. The best results are obtained using the PCA basis.

Moreover, the top two rows of Table 1 show that using the novel extension of our algorithm to colour images (mfsf c) described in Sect. 7 improves significantly the results in all versions of the sequence. Once more, the results obtained using a full rank PCA basis (mfsf *c* $_\mathtt{PCA}$) outperform those obtained with the DCT basis (mfsf *c* $_\mathtt{DCT}$).

Regarding the choice of parameters, as we described in Sect. 8 the default values proposed in Wedel et al. (2009) for the ITV-$\mathbf{L}^1$ algorithm were also found to give best results on our grayscale algorithm (mfsf). 8

However, we found that these parameters needed some tuning on the noisy and occluded versions of our benchmark sequence. A lower value of the data term weight $\alpha = 18$ was found to provide best results. Additionally, on the noisy sequences, the weight of the quadratic term was lowered to $\beta =0.4.$ These modified values were used on mfsf $_\mathtt{PCA}$, mfsf $_\mathtt{DCT}$and mfsf $_{\mathtt{I}_{2F}}$.

Figure 5 shows a visual comparison of the results on the benchmark sequence reported in Table 1. We show a closeup of the reverse warped images $\mathcal{W }^{-1}(I_{i})$ of three frames in the sequence ($i=20, 25, 30$) which should look identical to the template frame; and the error in the flow estimation $\epsilon _{i}$ for the same frames, expressed in pixels, encoded as a heatmap. Notice the significant improvements that our proposed algorithms for colour images (mfsf *c* $_\mathtt{PCA}$, mfsf *c* $_\mathtt{DCT}$) show with respect to their grayscale counterparts (mfsf $_\mathtt{PCA}$, mfsf $_\mathtt{DCT}$). Overall, all our approaches outperform state of the art methods: ITV-$\mathbf{L}^1$ optical flow (Wedel et al. 2009); LDOF (Brox and Malik 2011) and Pizarro and Bartoli’s registration algorithm (Pizarro and Bartoli 2010).

Figure 7 shows results of the experiments on the benchmark sequence with synthetic occlusions. The error maps $\epsilon _i$ for images ($i=20, 25, 30, 60$) encoded as heatmaps are shown for all the variants of our grayscale (mfsf $_\mathtt{PCA}$, mfsf $_\mathtt{DCT}$) and colour (mfsf *c* $_\mathtt{PCA}$, mfsf *c* $_\mathtt{DCT}$) algorithms as well as ITV-$\mathbf{L}^1$ (Wedel et al. 2009), LDOF (Brox and Malik 2011) and Pizarro and Bartoli (2010). We notice the same behaviour as in the experiments without occlusions—the error maps obtained with our algorithms show a superior performance with respect to state of the art approaches. Amongst our proposed approaches, one can observe significant improvements of the colour versions over their grayscale equivalents.

Figure 6a shows a graph of the RMS error over all the frames of the optical flow estimated using the 3 different bases for different values of the rank and of the weight $\beta$ associated with the soft constraint. For a reasonably large value of $\beta$ all the basis can be used with a significant reduction in the rank. The optimization also appears not to overfit when the dimensionality of the subspace is overly high. Figure 6c establishes the same fact in the case of noisy images and sequences with occlusions. Figure 6b explores the effect of varying the value of the weight $\beta$ on the accuracy of the optical flow. While low values of $\beta$ cause numerical instability (data and regularization terms become completely decoupled) high values of $\beta ,$ on the other hand, lead to slow convergence and errors since the point-wise search is not allowed to leave the manifold, simulating a hard constraint. Another interesting observation is that our proposed method with a PCA basis of rank $R$=50, yields a better performance than with a full rank PCA basis $R$=120. This reflects the fact that the temporal regularization due to the low dimensional subspace is often beneficial. Note that to analyze the sensitivity of our algorithm to its parameters in Fig. 6a–c we used ground truth tracks to compute the PCA basis to remove the bias from tracking.

### Experimental Comparison of Soft Versus Hard Subspace Constraint

In this section we use the synthetic grayscale flag sequence to conduct an experimental comparison of the optical flow obtained using our proposed soft subspace constraint with that obtained imposing the hard constraint described in Sect. 9. The energy associated with the hard constraint (59) can be obtained by removing the quadratic term $E_{link}$ from our energy (6) and reparameterizing the optical flow in terms of the trajectory coefficients.

We use the primal-dual algorithm described in Appendix C to minimise the energy obtained via reparameterization (59) with 200 iterations per warp to ensure convergence. We observed that 200 iterations were enough for the convergence of the cost function to a reasonable tolerance (which we consider to be when the change in cost per iteration is ${<}1000$th of the total change).

Our energy (6) based on the soft subspace constraint, is minimized using our optimization scheme described in Sect.5. To establish a fair comparison, we used 20 denoising iterations for the regularization step and 20 alternation iterations between the minimisation of Step 1 and Step 2 to ensure convergence.

Table 2 reports the RMS endpoint error, measured in pixels, of the flow obtained with the soft (S) and hard (H) constraints using 3 different basis:

1. Low rank ($R=75$) PCA basis obtained from sparse tracking using Pizarro and Bartoli (2010).
2. Full rank PCA basis obtained from ground truth optical flow.
3. Full rank DCT basis.

The comparative results in Table 2 show that the optical flow obtained with our soft constraint consistently outperforms the flow obtained after reparameterization (hard constraint) in all three experiments on all the different sequences (orginal, noisy and with occlusions). This is particularly the case in the presence of Gaussian noise when the endpoint errors differ most. However, this is to be expected since our soft constraint allows some deviations from the subspace manifold.

**Table 2:** RMS endpoint error in pixels for the optical flow obtained with the hard (H) versus soft (S) constraints

Basis	Rank	Constraint	Version of input sequence:			
			Original	Occl.	Gauss. noise	S&P noise
Sparse PCA	75	Soft (S)	**0.90**	**1.01**	**1.80**	**1.46**
		Hard (H)	0.98	1.05	2.22	1.60
GT PCA	120	Soft (S)	**0.69**	**0.76**	**1.43**	**1.07**
		Hard (H)	0.70	0.77	1.65	1.08
DCT	120	Soft (S)	**0.89**	**1.12**	**1.83**	**1.38**
		Hard (H)	1.09	1.28	2.00	1.42

We carry out 3 experiments using: (top) a low-rank sparse PCA basis (using tracks given by  Pizarro and Bartoli (2010)); (middle) a full rank ground truth PCA basis (computed using the ground truth optical flow); and (bottom) a full rank DCT basis. The algorithms were tested on all the different types of sequence (original, noisy and with occlusions)

In the first experiment we used a low rank PCA basis estimated from sparse tracking (obtained using Pizarro and Bartoli’s matching algorithm (Pizarro and Bartoli 2010)) to test the case of an inaccurate basis. This is the case when it is most clearly beneficial to allow deviations from the subspace manifold. This is naturally reflected on significantly higher endpoint errors on the flow computed with the hard constraint compared with that computed with our soft constraint.

It is also interesting to observe that even in the case when we used the full rank PCA basis computed from the ground truth flow the soft constraint performs marginally better than the hard constraint. In the sequence with Gaussian noise it provides a more clear benefit. Finally, the third experiment with a full rank DCT basis also shows that it is beneficial to use a soft constraint in all the different image sequences.

In conclusion, the optical flow obtained using the subspace constraint as a soft constraint consistently outperforms the flow obtained by reparameterization when both algorithms were ran until convergence. The benefits of the soft constraint are stronger when dealing with noisy images and in the case of an inaccurate motion basis which is to be expected.

### Experiments on Real Sequences

In this section we provide details about the experiments we have carried out on four video sequences which display large displacements and strong deformations.

#### Actor sequence

**Fig. 8:**
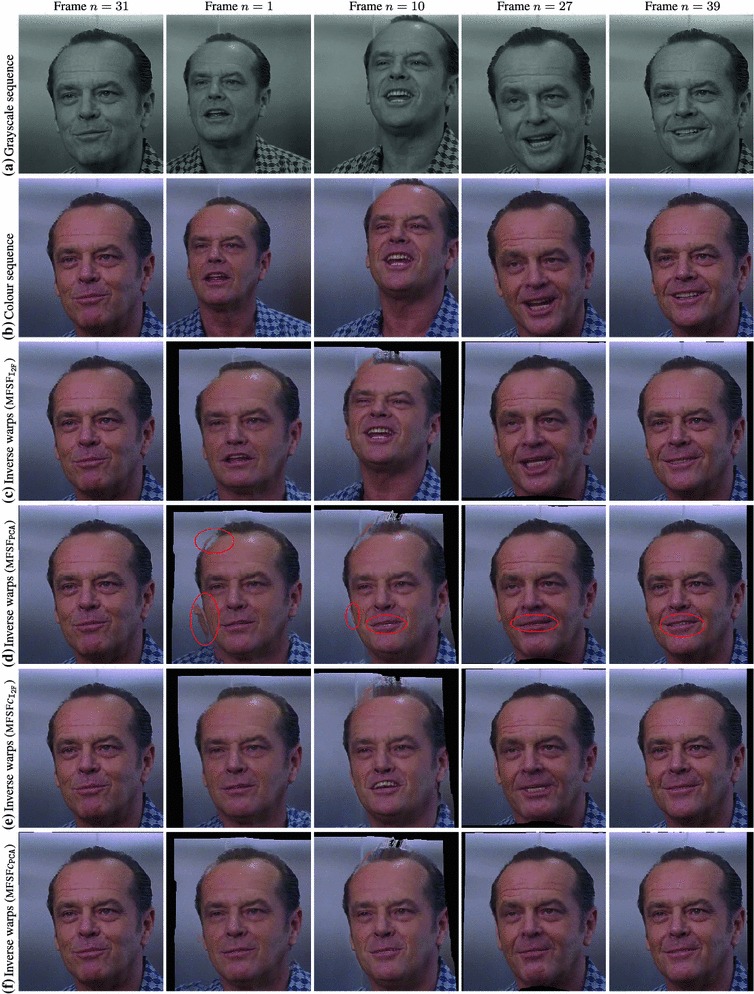
Results on the Actor sequence: (**a–b**) Some frames of the grayscale and colour input sequences. This is a challenging sequence with large displacements and strong deformations. Frame 31 $I_{31}$ is used as the reference frame. (**c–d**) Inverse warp images $\mathcal{W }^{-1}(I_n)$ comparing two versions of our grayscale algorithm: **c** without subspace constraints (mfsf $_{\mathtt{I}_{2F}}$) and (**d**) with subspace constraints (mfsf *c* $_\mathtt{I_{2F}}$). (**e–f**) Inverse warp images $\mathcal{W }^{-1}(I_n)$ comparing two versions of our colour algorithm: (**e**) without subspace constraints (mfsf *c* $_\mathtt{I_{2F}}$) and (**f**) with subspace constraints (mfsf *c* $_\mathtt{PCA}$)

This challenging sequence is a 39 frame long clip from a well known film, acquired at $25$ frames per second with images of size $500\times 550$ pixels. The top two rows of Fig. 8 show $5$ frames of this sequence in grayscale and colour. Note that frame $31$ was used as the reference frame 9. The bottom four rows in Fig. 8 show comparative results of the inverse warp images (using the computed optical flow to warp the current image back to the reference frame) estimated using the following different versions of our algorithm: mfsf $_{\mathtt{I}_{2F}}$, mfsf $_\mathtt{PCA}$, mfsf *c* $_\mathtt{I_{2F}}$, mfsf *c* $_\mathtt{PCA}$. The first two methods work on grayscale images and use the identity matrix and PCA basis as the motion basis respectively while the last two are their equivalent colour versions. Comparing the results of mfsf $_{\mathtt{I}_{2F}}$and mfsf $_\mathtt{PCA}$(or mfsf *c* $_\mathtt{I_{2F}}$and mfsf *c* $_\mathtt{PCA}$) allows us to show the advantages of using subspace constraints (PCA basis) versus not using a temporal model for the trajectories ($I_{2F}$ basis). We use a full rank PCA basis obtained after applying principal components analysis to an initial flow estimated with our algorithm using the DCT basis.

The advantages of using subspace constraints are clear. For instance, notice that for grayscale images mfsf $_{\mathtt{I}_{2F}}$ failed completely to warp frame $10$ while mfsf $_\mathtt{PCA}$ provides an accurate inverse warp image for the same frame and consistently superior results throughout the sequence. It is also clear that making use of all three colour channels using the extension of our algorithm to vector valued images provides substantial improvements. Both mfsf *c* $_\mathtt{I_{2F}}$ and mfsf *c* $_\mathtt{PCA}$ outperform their grayscale equivalents. In row (d) of Fig. 8 we have highlighed in red areas where the flow has clearly failed on the grayscale mfsf $_\mathtt{PCA}$algorithm but have been correctly warped in its colour version mfsf *c* $_\mathtt{PCA}$ .

Notice also that mfsf *c* $_\mathtt{I_{2F}}$copes with the large displacements in frame $10$ much better than mfsf $_{\mathtt{I}_{2F}}$. However, just using colour without subspace constraints is not enough to estimate accurate flow. Comparing the bottom two rows of Fig. 8 reveals that using subspace constraints significantly improves results also in the case of colour. In conclusion, the best overall results are obtained with mfsf *c* $_\mathtt{PCA}$, our colour algorithm with subspace constraints using the PCA basis.

**Fig. 9:**
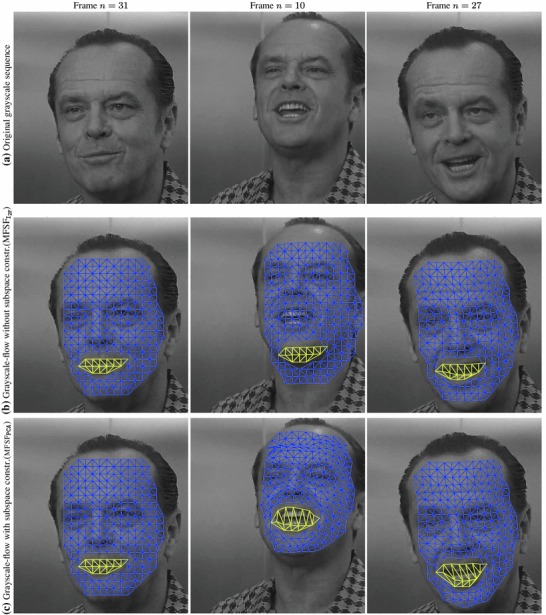
Results on the grayscale Actor sequence: *Top row* (**a**) shows some frames of the original grayscale sequence. *Middle* (**b**) and *bottom* (**c**) rows compare the optical flow results obtained with two of our proposed grayscale algorithms: (**c**) with subspace constraints (mfsf $_\mathtt{PCA}$) and (**b**) without subspace constraints (mfsf $_{\mathtt{I}_{2F}}$). The flow is visualized with a grid superimposed on the images to reveal the optical flow in a sparse subset of points. Points on the mouth are shown in *yellow* to highlight the results on the area with strongest deformations (Color figure online)

**Fig. 10:**
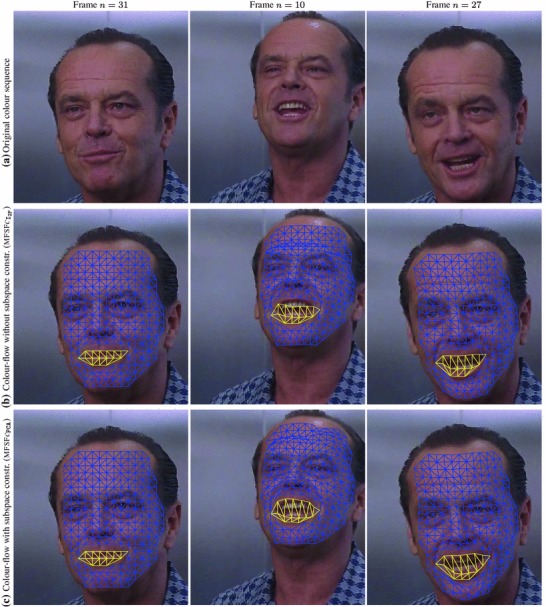
Results on the colour Actor sequence: *Top row* (**a**) shows some frames of the original colour sequence. *Middle* (**b**) and *bottom* (**c**) rows compare the optical flow results obtained with two of our proposed colour algorithms: (**c**) with subspace constraints (mfsf *c* $_\mathtt{PCA}$) and (**b**) without subspace constraints (mfsf *c* $_\mathtt{I_{2F}}$). The flow is visualized with a grid superimposed on the images to reveal the optical flow in a sparse subset of points. Points on the mouth are shown in *yellow* to highlight the results on the area with strongest deformations (Color figure online)

Figures 9 and 10 support our claims by showing a grid superimposed on the images to reveal the optical flow in a sparse subset of points. The points on the mouth are highlighted in yellow since that is where most of the deformations occur. Once more, Fig. 9 reveals that the quality of the flow computed using trajectory regularization constraints on grayscale images (mfsf $_\mathtt{PCA}$) is far better than that obtained without using subspace constraints (mfsf $_{\mathtt{I}_{2F}}$). Notice the complete failure of mfsf $_{\mathtt{I}_{2F}}$on frame $10.$ Similar conclusions can be drawn from the results on the colour images shown in Fig.10. Notice the improvements particularly on the lips.

#### Actress sequence

**Fig. 11:**
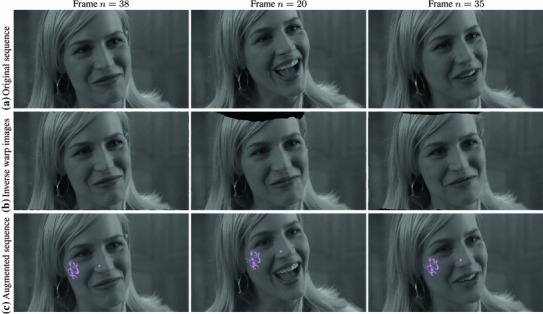
Results on the Actress sequence: (**a**) Some frames of the original grayscale sequence. (**b**) Inverse warp images obtained with our best performing grayscale method using subspace constraints (mfsf $_\mathtt{PCA}$). (**c**) Original images augmented with some texture to simulate a tattoo

This $72$ frame long clip from the same film shows a close-up of an actress opening the mouth widely. The resolution of the images was $640\times 360$ pixels. This sequence is similarly challenging to the previous one with very large displacements and deformations. In this case we only ran our best performing method on grayscale images mfsf $_\mathtt{PCA}$with subspace constraints using a PCA basis of rank $R=100.$ Figure 11 shows the original sequence (top row); the inverse warp images estimated from the optical flow (middle row) and the original images augmented with some texture (bottom row) to simulate a tattoo.

#### Paper bending-1 sequence

**Fig. 12:**
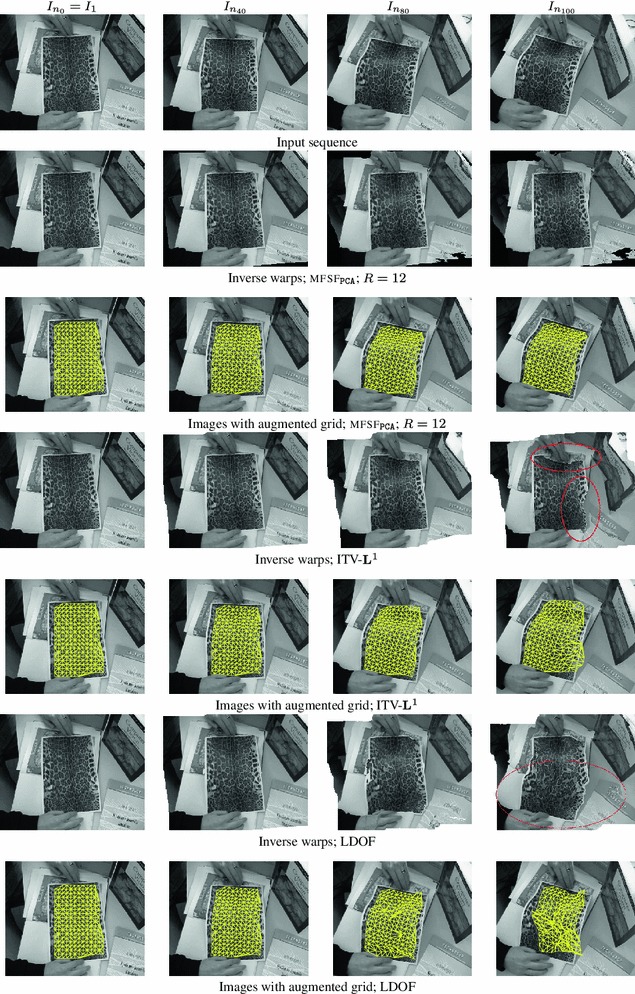
Results on Paper Bending-1 grayscale sequence: Comparativev results of the optical flow estimated with our best performing grayscale algorithm (mfsf $_\mathtt{PCA}$) against state of the art optical flow methods (ITV-$\mathbf{L}^1$(Wedel et al. 2009), LDOF (Brox and Malik 2011)). We show two visualizations of the optical flow estimated with the three methods in alternate rows: (i) the inverse warped images and (ii) a grid superimposed on the images to reveal the optical flow in a sparse subset of points. *Top row* shows some frames of the original sequence

Figure 12 shows results on a sequence of textured paper bending smoothly  (Bartoli et al. 2008); a challenging sequence due to its length ($100$ frames) and the large camera rotation. We show results comparing our best performing grayscale algorithm (mfsf $_\mathtt{PCA}$) against state of the art optical flow methods (ITV-$\mathbf{L}^1$ (Wedel et al. 2009), LDOF (Brox and Malik 2011)). For completeness in our experimental evaluation, in this case we computed the motion basis by applying PCA to KLT tracks (Lucas and Kanade 1981) keeping the first 12 components. We ran the LDOF and ITV-$\mathbf{L}^1$ algorithms using a multi-resolution scaling factor of 0.95, whereas for our algorithm the value 0.75 was sufficient (pointing to faster convergence). Comparing the warped images $\mathcal{W }^{-1}(I_{n}),$ we observe that our method yields a significant improvement on the accuracy of the optical flow, especially after some frames (see e.g. the artifacts annotated by the red ellipses in the results of LDOF and ITV-$\mathbf{L}^1$). We show an alternative visualization of the same results with a grid superimposed on the images to reveal the optical flow in a sparse subset of points. This visualization helps to highlight the superiority of the optical flow estimated with our algorithm (mfsf $_\mathtt{PCA}$) with respect to others.

**Fig. 13:**
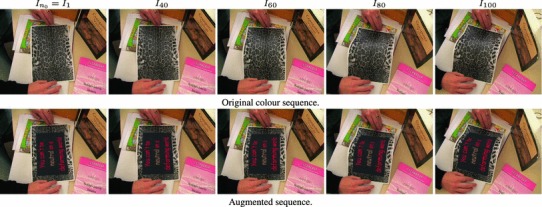
Results on Paper Bending-1 colour sequence: The *top row* shows some frames of the original colour sequence. The *bottom row* displays the same sequence augmented with some new texture. The optical flow obtained with our best performing colour algorithm mfsf *c* $_\mathtt{PCA}$was used to re-texture the original sequence

In Fig. 13 we show results on the colour version of this sequence, subsampled taking every fifth frame to give a $25$ frame long sequence. In this case, we augment the images with new texture using the optical flow results given by our colour multi-frame subspace algorithm using a PCA basis (mfsf *c* $_\mathtt{PCA}$). In this case we use a full rank PCA basis obtained after applying principal components analysis to an initial flow estimated with our algorithm using the DCT basis (mfsf *c* $_\mathtt{DCT}$).

#### Paper bending-2 sequence

**Fig. 14:**
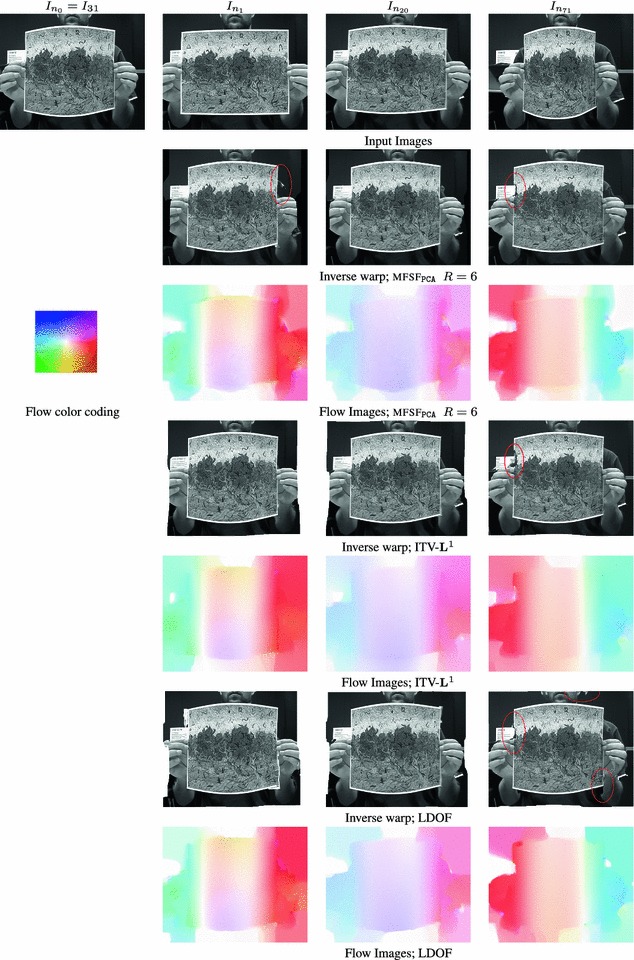
Results on the Paper bending-2 sequence: *Top row* shows some images of this grayscale sequence. The 30th frame is used as the reference. Next rows show inverse warp images and colour coded optical flow comparing our best performing grayscale algorithm (mfsf $_\mathtt{PCA}$) using a very low rank PCA decomposition ($R=6$) against state of the art optical flow methods (ITV-$\mathbf{L}^1$ (Wedel et al. 2009), LDOF (Brox and Malik 2011))

Figure 14 shows a $71$ frame long grayscale sequence introduced in Varol et al. (2009) of a paper being bent backwards which is widely used for 3D reconstruction in non-rigid structure from motion (NRSfM). Our method used a PCA basis of rank $R=6$ obtained from KLT tracks. The $30$th frame is used as the reference. Once more, we compare results of our algorithm (mfsf $_\mathtt{PCA}$) against the same state of the art approaches as in previous experiments. The inverse warped images and the colour coded optical flow in Fig. 14 reveal that despite having used a very low rank PCA motion basis, our results outperform LDOF and provide more accurate flow boundaries than ITV-$\mathbf{L}^1.$

## Conclusions

We have provided a new formulation for the computation of multi-frame optical flow exploiting the high correlation between 2D trajectories of points in a long sequence by assuming that these lie close to a low dimensional subspace. Our main contribution is to formulate the manifold constraint as a *soft constraint* which, using variational principles, leads to a *robust* energy that can be efficiently optimized. We propose a new *anisotropic trajectory regularization* term that acts on the coefficients of the trajectory basis. We take advantage of the high level of parallelism inherent to our approach by developing a GPU implementation using the Nvidia CUDA framework. We also provide an extension of our approach to the case of vector-valued images which allows us to exploit all three colour channels and gain substantial improvements in the accuracy of the estimated optical flow. We also provide a new benchmark dataset, with ground truth optical flow. Our experimental results on the benchmark dataset and on real video footage reveal that using subspace constraints significantly improves results. Our approach outperforms state of the art optical flow and non-rigid registration algorithms.

## Appendix A: Primal Dual Algorithm for Denoising

This appendix describes the optimization of the energy minimized in Step 1 of our algorithm as defined in (22):

36 $$\begin{aligned} E_d = \int _\Omega \, g(\varvec{x}) \left|\nabla L(\varvec{x}) \right|_\epsilon + \beta (M(\varvec{x}) -L(\varvec{x})) ^2 \mathrm{d}\varvec{x}\end{aligned}$$

which corresponds to a small modification of the TV-$\mathbf{L}^2$ Rudin-Osher-Fatemi (ROF) model (Rudin et al. 1992), as described in Sect. 5.1. Note that as the trajectory model coefficients $L_i(\varvec{x})$ in (22) are decoupled for each $i,$ in the following derivation we have dropped the subscript for simplicity.

The first step in the optimization is the dualisation of the weighted Huber functional $g(x) H_\epsilon (|\nabla L(\varvec{x}) |^2)$ of the above energy with respect to the gradient $\nabla L (\varvec{x})$ using its Legendre-Fenchel transform (Rockafellar 1997). After spatial discretization, the minimisation of (36) is equivalent to the following saddle point problem:

37 $$\begin{aligned} \min _{\varvec{L}} \max _{\varvec{\mathcal{L }}}\sum _{\varvec{x}\in \varvec{X}}&\left\{ \langle \nabla L(\varvec{x}),\varvec{\mathcal{L }}(\varvec{x})\rangle - \delta \left( \frac{\varvec{\mathcal{L }}(\varvec{x})}{g( \varvec{x})}\right) -\epsilon \frac{|\varvec{\mathcal{L }}(\varvec{x})|^2}{2g(\varvec{x})}\right. \nonumber \\&\left. + \beta (M(\varvec{x}) -L(\varvec{x})) ^2 \right\} . \end{aligned}$$

where $\varvec{X}$ is the set of image grid points, $\nabla$ denotes the discrete gradient operator as defined in Chambolle and Pock (2011), $\varvec{\mathcal{L }}(\varvec{x}) \in \mathbb{R }^{2}$ are the dual variables for every $(\varvec{x}),$ and $\delta (\varvec{\mathcal{L }})$ is the indicator function of the unit ball:

38 $$\begin{aligned} \delta (\varvec{\mathcal{L }}) \triangleq \left\{ \begin{array}{ccc} 0 &{}  \text{ if } \ &{} \left|\varvec{\mathcal{L }}\right|\le 1 \\ \infty &{}  \ &{} \text{ otherwise } \end{array} \right. \end{aligned}$$

The problem (37) can be considered as a special case of the following general form of primal-dual problems that are studied in Chambolle and Pock (2011):

39 $$\begin{aligned} \min \limits _{p} \max \limits _{q} \langle Kp,q \rangle -F^{*}(q) + G(p). \end{aligned}$$

In the case of (37), the norm of the linear operator $K = \nabla$ is bounded by $\sqrt{8}.$ Also, both $G$ and $F^{*}$ are uniformly convex with convexity parameters $2\beta$ and $\epsilon$ respectively.

Therefore, we solve (37) by applying Algorithm 3 of  Chambolle and Pock (2011). The steps of the algorithm can be written as follows :

- Initialize $$\varvec{\mathcal{L }}^0=\varvec{0}, L^0(\varvec{x}) = \bar{L}^0(\varvec{x}) = M(x)$$
- Iterate for $k=0,1,2,\ldots$ until a convergence criterion is satisfied:

40 $$\begin{aligned} \varvec{\mathcal{L }}^{k+1}(\varvec{x})&= g(\varvec{x}) \Pi \left( \frac{\varvec{\mathcal{L }}^{k}(\varvec{x}) + \tau \nabla \bar{L}^{k}(\varvec{x}) }{g(\varvec{x}) + \tau \epsilon } \right) \end{aligned}$$

41 $$\begin{aligned} L^{k+1}(\varvec{x})\!&\!=\!&\! \frac{1}{1\!+\! 2\sigma \beta } \left( 2\sigma \beta M(\varvec{x}) \!+\!L^{k}(\varvec{x})\!+\!\sigma div (\varvec{\mathcal{L }}^{k+1}(\varvec{x})) \right) \end{aligned}$$

42 $$\begin{aligned} \bar{L}^{k+1}(\varvec{x})&= 2 L^{k+1}(\varvec{x}) - L^{k}(\varvec{x}) \end{aligned}$$

where $div(.)$ is the descrete divergence operator and the operator $\Pi (\varvec{s})$ projects a vector $\varvec{s}$ onto the unit ball as:

43 $$\begin{aligned} \Pi (\varvec{s}) = \frac{\varvec{s}}{\max (1,|\varvec{s}|)} \end{aligned}$$

We choose the following values for the steps $\sigma ,\tau ,$ that guarantees the convergence:

44 $$\begin{aligned} \sigma = \sqrt{\frac{\epsilon }{16\beta }}, \tau = \sqrt{\frac{\beta }{4\epsilon }} \end{aligned}$$

## Appendix B: Primal Dual Algorithm for Robust Vector-Valued Image Matching

This appendix provides the details of the algorithm to optimise the saddle point problem (32) for vector-valued images using Euclidean norm and Huber penalisers.

### Euclidean Norm Penaliser

This case corresponds to $\Phi (\varvec{v})=|\varvec{v}|$ and is a straightforward extension of the absolute value of image differences that we used for $E_{data}$ in (7) for grayscale images. After dualisation, (32) can be written as:

45 $$\begin{aligned} \min \limits _{\varvec{u}} \max \limits _{\varvec{\mathcal{I }}} \left\{ \langle \alpha \varvec{A}\varvec{u}, \varvec{\mathcal{I }}\rangle + \alpha \langle \varvec{b},\varvec{\mathcal{I }}\rangle - \delta (\varvec{\mathcal{I }}) + \beta \left|\varvec{u}- \varvec{u}^{\prime } \right|^2 \right\} \end{aligned}$$

This problem is also a special case of the general saddle point problem (39) with the linear operator $K = \alpha \varvec{A}.$ Since the function $\beta \left|\varvec{u}- \varvec{u}^{\prime } \right|^2$ is uniformly convex with convexity parameter $2\beta ,$ we apply Algorithm 2 of  Chambolle and Pock (2011) and derive following optimisation algorithm:

- Choose $$\sigma _0 = \tau _0 = \frac{1}{\alpha B_A}$$
- Initialize $\varvec{u}^0$ from the previous alternation iteration.
- Initialize $$\bar{\varvec{u}}^0 = \varvec{u}^0, \varvec{\mathcal{I }}^{k} = \varvec{0}.$$
- Iterate for $k=0,1,2,\ldots$ until a convergence criterion is satisfied:
  46 $$\begin{aligned} \varvec{\mathcal{I }}^{k+1}&= \Pi (\varvec{\mathcal{I }}^{k} +\alpha \tau _k(\varvec{A}\bar{\varvec{u}}^{k} + \varvec{b}))\end{aligned}$$
  47 $$\begin{aligned} \varvec{u}^{k+1}&= \frac{1}{1+2\sigma _k \beta } \left( 2\sigma _k \beta \varvec{u}^{\prime } + \varvec{u}^k - \sigma _k \varvec{A}^T \varvec{\mathcal{I }}^{k+1} \right) ,\end{aligned}$$
  48 $$\begin{aligned} \theta _k&= \frac{1}{\sqrt{1+4\beta \sigma _k}}, \ \ \sigma _{k+1} = \theta _k\sigma _k, \ \ \ \tau _{k+1} = \frac{\tau _k}{\theta _k} \end{aligned}$$
  49 $$\begin{aligned} \bar{\varvec{u}}^{k+1}&= \varvec{u}^{k+1} + \theta _k (\varvec{u}^{k+1}-\varvec{u}^{k}) \end{aligned}$$

where $B_A$ can be any upper bound on the norm of $\varvec{A}.$ Although the saddle point problem is minimised separately for each spatio-temporal point of the video and $\varvec{A}$ is spatially varying, for simplicity we choose a common upper bound on the linear operator for all the points. It can be shown that $L_A$ as defined below is a valid upper bound.

50 $$\begin{aligned} B_A \!=\! \sqrt{ \max _{n} \sum _{i = 1}^{N_c}\left( \max _{\varvec{x}} \left| \frac{\partial \varvec{I}_i(\varvec{x};n)}{\partial x}\right| ^2 \!+\! \max _{\varvec{x}} \left| \frac{\partial \varvec{I}_i(\varvec{x};n)}{\partial y}\right| ^2 \right) }\nonumber \\ \end{aligned}$$

where $(x,y)$ are the horizontal and vertical coordinate axes of the image plane.

### Huber Penaliser

When the robust function used in the data term of the energy for vector-valued images is the Huber norm: $\Phi (\varvec{v})=H_\epsilon (|\varvec{v}|^2),$ the saddle point problem (32) can be written as:

51 $$\begin{aligned} \min \limits _{\varvec{u}} \max \limits _{\varvec{\mathcal{I }}} \left\{ \langle \varvec{A}\varvec{u}, \varvec{\mathcal{I }}\rangle \!+\! \langle \varvec{b},\varvec{\mathcal{I }}\rangle \!-\! \frac{\epsilon }{2 \alpha } \left|\varvec{\mathcal{I }}\right|^2 \!-\! \delta \left( \frac{\varvec{\mathcal{I }}}{\alpha }\right) \!+\! \beta \left|\varvec{u}- \varvec{u}^{\prime } \right|^2 \right\} \nonumber \\ \end{aligned}$$

This problem is again of the form (39) with the linear operator $K =\varvec{A}.$ The corresponding $G$ and $F^{*}$ functions are both uniformly convex with parameters $2\beta$ and $\frac{\epsilon }{\alpha }.$ We thus solve (51) using Algorithm 3 of  Chambolle and Pock (2011) and derive the following optimisation algorithm:

- Initialize $\varvec{u}^0$ from the previous alternation iteration.
- Initialize $$\bar{\varvec{u}}^0 = \varvec{u}^0, \varvec{\mathcal{I }}^{k} = \varvec{0}.$$
- Iterate for $k=0,1,2,3,\ldots$ until a convergence criterion is satisfied:
  52 $$\begin{aligned} \varvec{\mathcal{I }}^{k+1}&= \alpha \Pi \left( \frac{\varvec{\mathcal{I }}^{k} + \tau (\varvec{A}\bar{\varvec{u}}^{k} + \varvec{b})}{\alpha +\tau \epsilon }\right) \end{aligned}$$
  53 $$\begin{aligned} \varvec{u}^{k+1}&= \frac{1}{1+2\sigma \beta } \left( 2\sigma \beta \varvec{u}^{\prime } + \varvec{u}^k - \sigma \varvec{A}^T \varvec{\mathcal{I }}^{k +1}\right) ,\end{aligned}$$
  54 $$\begin{aligned} \bar{\varvec{u}}^{k+1}&= 2\varvec{u}^{k+1} -\varvec{u}^{k} \end{aligned}$$

We choose the following step-sizes which ensure the convergence of our algorithm:

55 $$\begin{aligned} \sigma = \frac{1}{B_A }\sqrt{\frac{\epsilon }{2\beta \alpha }}, \tau = \frac{1}{B_A }\sqrt{\frac{2\beta \alpha }{\epsilon }} \end{aligned}$$

where $B_A$ is, again, any upper bound on the operator norm of $\varvec{A}.$ As in the case of Euclidean norm penalisation, we choose $B_A$ as defined in (50).

## Appendix C: Optimization of the Hard Subspace Constraint

This appendix describes the optimization of the energy

56 $$\begin{aligned} E_{h}&= \int _\Omega \sum _{n=1}^F \left| \varvec{I}\left( \varvec{x}+ Q_n \varvec{L}(\varvec{x}) \,\,;\,\, n \right) - \varvec{I}(\varvec{x}; n_0) \right| \, \mathrm{d}\varvec{x}\nonumber \\&+ \int _\Omega \sum _{i=1}^R \,\,g(\varvec{x}) \left|\nabla L_i(\varvec{x}) \right|_\epsilon \, \mathrm{d}\varvec{x}\end{aligned}$$

which corresponds to the case when the subspace constraint is imposed as a hard constraint and the 2D flow $\varvec{u}(\varvec{x};n)$ can be reparameterized as $\sum \limits _{i=1}^R \varvec{q}_i(n) L_i(\varvec{x}).$ First, each image channel of $\varvec{I}(\cdot ;n)$ is linearised around $Q_n\varvec{L}_0(\varvec{x}),$ using an initial estimate $\varvec{L}_0(\varvec{x}).$ Under this approximation the data term can be written as:

57 $$\begin{aligned} E_{data} = \int _\Omega \sum _{n=1}^F \left| {\varvec{C}}(\varvec{x};n) \varvec{L}(\varvec{x}) + \varvec{d}(\varvec{x};n) \right| \, \mathrm{d}\varvec{x}\end{aligned}$$

where, for every spatio-temporal point $(\varvec{x};n),$

58 $$\begin{aligned} {\varvec{C}}(\varvec{x};n) = \frac{\partial \varvec{I}\left( \varvec{x}+ Q_n \varvec{L}_0(\varvec{x}) \,\,;\,\, n \right) }{\partial \varvec{x}} Q_n \end{aligned}$$

is the $N_c\times R$ Jacobian matrix and $\varvec{d}(\varvec{x};n) = \varvec{I}(\varvec{x}+Q_n \varvec{L}_0(\varvec{x});n)- \varvec{I}(\varvec{x}; n_0)$ $-{\varvec{C}}(\varvec{x};n) \varvec{L}_0(\varvec{x})$ is a $N_c$ dimensional vector.

Thus, the following minimization problem must be solved:

59 $$\begin{aligned} \min _{\varvec{L}(\varvec{x})} \int _\Omega \left\{ \alpha \sum _{n=1}^F \left| \rho (\varvec{L}(\varvec{x});n) \right| + \sum _{i=1}^R \,g(\varvec{x}) \left|\nabla L_i(\varvec{x}) \right|_\epsilon \right\} \mathrm{d}\varvec{x}.\nonumber \\ \end{aligned}$$

where $\rho (\varvec{L}(\varvec{x});n) = {\varvec{C}}(\varvec{x};n) \varvec{L}(\varvec{x}) + \varvec{d}(\varvec{x};n)$ is the linearised color constancy. After dualisation of the data and regularisation terms and spatial discretization, the minimisation (59) is equivalent to the following saddle point problem:

60 $$\begin{aligned} \min _{\varvec{L}}\!\!\!\!&\max _{\varvec{\mathcal{I }},\varvec{\mathcal{L }}}\sum _{\varvec{x}\in \varvec{X}} \left\{ \alpha \sum _{n=1}^F \big ( \langle \rho (\varvec{L}(\varvec{x});n), \varvec{\mathcal{I }}(\varvec{x};n)\rangle \!-\! \delta (\varvec{\mathcal{I }}(\varvec{x};n)) \big ) \right. \nonumber \\ \!+\! \left. \sum _{i=1}^R \left( \langle \nabla L_i(\varvec{x}),\varvec{\mathcal{L }}_i(\varvec{x}) \rangle \!-\! \delta \left( \frac{\varvec{\mathcal{L }}_i(\varvec{x})}{g(\varvec{x})}\right) \!-\! \epsilon \frac{|\varvec{\mathcal{L }}_i(\varvec{x})|^2}{2g(\varvec{x})} \right) \right\} \nonumber \\ \end{aligned}$$

where $\varvec{\mathcal{I }}(\varvec{x};n) \in \mathbb{R }^{N_c}$ and $\varvec{\mathcal{L }}_i(\varvec{x}) \in \mathbb{R }^{2}$ are the dual variables for every $(\varvec{x};n)$ and $(\varvec{x};i)$ respectively.

The energy (60) can be considered as a special case of the general form of primal-dual problem (39) where the linear operator $K$ is the ${(N_cF + 2R)N_p \times RN_p}$ dimensional matrix:

61 $$\begin{aligned} K= \left[ \begin{array}{c} \alpha \ \tilde{\mathbf{C}} \\ \varvec{\nabla } \end{array} \right] ; \ \ \tilde{\mathbf{C}} = \left[ \begin{array}{ccc} \begin{array}{c} {\varvec{C}}(\varvec{x}_1;1) \\ \vdots \\ {\varvec{C}}(\varvec{x}_1;F) \end{array} &{}&{}\\ &{}\ddots &{} \\ &{}&{}{\begin{array}{c} {\varvec{C}}(\varvec{x}_{N_p};1) \\ \vdots \\ {\varvec{C}}(\varvec{x}_{N_p};F) \end{array}} \end{array} \right] \end{aligned}$$

where $\varvec{x}_1, \ldots , \varvec{x}_{N_p}$ are the image grid points and $n \in \{1,\cdots ,F\}.$

Thus, we solve (52) by applying Algorithm 1 of  Chambolle and Pock (2011). In this case, the steps of this algorithm can be written as follows :

- Initialize $$\varvec{L}^0(\varvec{x}) = \bar{\varvec{L}}^0(\varvec{x}) = \varvec{L}_0(\varvec{x})$$
- Initialize $$\varvec{\mathcal{I }}^0(\varvec{x};n)=\varvec{\mathcal{L }}^0(\varvec{x};n)=\varvec{0}$$
- Iterate for $k=0,1,2,\ldots$ until a convergence criterion is satisfied:

62 $$\begin{aligned} \varvec{\mathcal{I }}^{k+1}(\varvec{x};n)&= \Pi \left( \varvec{\mathcal{I }}^{k}(\varvec{x};n) + \tau \alpha \rho (\bar{\varvec{L}}^{k}(x);n) \right) \end{aligned}$$

63 $$\begin{aligned} \varvec{\mathcal{L }}_i^{k+1}(\varvec{x})&= g(\varvec{x}) \Pi \left( \frac{\varvec{\mathcal{L }}_i^{k}(\varvec{x}) + \tau \nabla \bar{L}_i^{k}(\varvec{x}) }{g(\varvec{x}) + \tau \epsilon } \right) \end{aligned}$$

64 $$\begin{aligned} L_i^{k+1}(\varvec{x})&= L_i^{k}(\varvec{x}) - \sigma \alpha \sum _{n=1}^F C(\varvec{x};n)^ T \varvec{\mathcal{I }}^{k+1}(\varvec{x};n)\nonumber \\&+\,\sigma div (\varvec{\mathcal{L }}_i^{k+1}(\varvec{x})) \end{aligned}$$

65 $$\begin{aligned} \bar{\varvec{L}}^{k+1}(\varvec{x})&= 2 \varvec{L}^{k+1}(\varvec{x}) - \varvec{L}^{k}(\varvec{x}) \end{aligned}$$

We use the following step-sizes, which guarantee the convergence of this algorithm too:

66 $$\begin{aligned} \sigma = \tau = \frac{1}{B_K} \end{aligned}$$

$B_K$ is the following upper bound on the operator norm of $K$ (61):

67 $$\begin{aligned} B_K = \sqrt{ 8+ \alpha ^2 B_A^2 } \end{aligned}$$

where $B_A$ is given by (50).

## Footnotes

1 The parametric warp functions used in this work include Thin Plate Spline (TPS) and Free-Form Deformations (FFD) based on 2D Cubic B-Splines.

2 After the linearization of the brightness constancy term

3 In Weickert and Schnörr (2001a) this design principle is expressed for the classical optical flow case where the input is a single pair of frames, but here we present its straight-forward extension to the case of multiple frames.

4 By *specific reference point* we mean that we associate the new location (after rotation) of a point on the reference image with its original location.

5 Note that, for the sake of clarity in our presentation, the generic robust function $\Phi$ defined here differs from the robust function $\Psi$ that we used in Sect. 4: $\Phi$ is applied directly to the vectorial differences whereas $\Psi$ is applied to their squared norms. The two definitions are linked by: $\Phi (\varvec{v})=\Psi (|\varvec{v}|^2)$

6 Note that we have normalized the image intensity values to lie between $0$ and $1.$

7 Videos of the results as well as our benchmark dataset can be found on the following URL: http://www.eecs.qmul.ac.uk/~lourdes/subspace_flow

8 Note that, as we discussed in Sect. 4, mfsf $_{\mathtt{I}_{2F}}$ and ITV-$\mathbf{L}^1$  Wedel et al. (2009) are equivalent algorithms and should therefore provide the same results. The difference in the numerical results is due to two factors: (i) in mfsf $_{\mathtt{I}_{2F}}$ $c_g=0$ and $\epsilon \ne 0$ (ii) the ITV-$\mathbf{L}^1$ algorithm was run with its default parameters and mfsf $_{\mathtt{I}_{2F}}$ with the tuned parameters described above.

9 We choose the reference frame to be one in which the points we are interested in tracking are all visible and also to reduce the maximum displacements.
